# Myoprotective Whole Foods, Muscle Health and Sarcopenia: A Systematic Review of Observational and Intervention Studies in Older Adults

**DOI:** 10.3390/nu12082257

**Published:** 2020-07-28

**Authors:** Antoneta Granic, Lorelle Dismore, Christopher Hurst, Sian M. Robinson, Avan A. Sayer

**Affiliations:** 1 AGE Research Group, Translational and Clinical Research Institute, Newcastle University, Newcastle upon Tyne NE1 7RU, UK; antoneta.granic@newcastle.ac.uk (A.G.); lorelle.dismore@newcastle.ac.uk (L.D.); christopher.hurst@newcastle.ac.uk (C.H.); sian.robinson@newcastle.ac.uk (S.M.R.); 2 NIHR Newcastle Biomedical Research Centre, Newcastle upon Tyne Hospitals NHS Foundation Trust and Newcastle University, Newcastle upon Tyne NE4 5PL, UK; 3 Northumbria Healthcare NHS Foundation Trust, Research and Development, North Tyneside General Hospital, North Shields NE29 8NH, UK

**Keywords:** myoprotective, whole foods, muscle function, sarcopenia, older adults, observational studies, intervention studies

## Abstract

Decline in skeletal muscle strength and mass (sarcopenia) accelerates with age, leading to adverse health outcomes and poor quality of life. Diet plays a crucial role in muscle ageing being an important element of a healthy lifestyle. However, unlike single nutrients, such as dietary protein, or dietary patterns, such as the Mediterranean diet, the relationship between individual whole foods and muscle health has not been systematically evaluated. We aimed to investigate which whole foods (meat, fish, eggs, fruit and vegetables, and non-liquid dairy) may be beneficial (myoprotective) for ageing muscle and sarcopenia in adults aged ≥ 50 years. Nineteen observational and nine intervention studies were identified through systematic searches of the four electronic databases (last search: March 2020). The synthesis of findings showed strong and consistent evidence for a beneficial effect of lean red meat on muscle mass or lean tissue mass in both observational and intervention studies. Higher intake of fruit and vegetables was associated with better muscle function in observational studies, but the evidence from intervention studies was scarce. Non-liquid dairy foods were beneficial for muscle mass in both observational and intervention studies. There was moderate evidence for the role of these foods in muscle strength and sarcopenia, and limited or inconclusive evidence for the benefits of other whole foods (e.g., fish, eggs) for muscle health in older adults. Although current nutritional recommendations are often based on a single nutrient approach, further research about the role of protein-rich and other foods in muscle health will allow for the development of guidelines that are based on whole foods, also highlighting the potential importance of non-protein nutrients within these foods for myoprotection in older adults.

## 1. Introduction

Loss of skeletal muscle strength and mass (sarcopenia; from Greek *sarx* “flesh” and *penia* “poverty”) [1,2] accelerates with advancing age [3], and contributes to adverse health outcomes in older adults, increasing the risk of disability, poor quality of life, hospitalisation, and death [1,2,3,4,5]. A recent systematic review of studies estimating the economic burden-of-illness has shown major healthcare costs of sarcopenia, especially related to hospitalisation of sarcopenic versus non-sarcopenic patients [6]. Thus, finding cost-effective prevention and treatment for sarcopenia is of great societal and public health interest. However, despite recent improvements in defining sarcopenia [1,2] and understanding disease aetiology [2], challenges remain in how to diagnose and treat sarcopenia in clinical practice [1,2], and prevent muscle health decline in the general population.

Current evidence implicates modifiable lifestyle factors, diet and exercise, as non-pharmacological treatments for poor muscle health and sarcopenia [7,8,9,10,11,12,13,14,15,16,17,18], implemented either alone [10,12,13,15] or in combination [9,11]. Several approaches have been utilised to investigate the diet-muscle relationship with ageing, employing either a single nutrient [9,10,11,12,13], whole food [14,15,16], or whole diet approach [17,18], whilst resistance exercise (RE) has been recognised as a powerful stimulus for muscle anabolism in older adults [19]. Specifically, interventions with protein supplements and RE have been effective in ameliorating the decline in muscle mass and function in older adults with and without sarcopenia and frailty [7,10]. Furthermore, several recent systematic reviews exploring the role of diet and dietary patterns have shown positive association between components of sarcopenia and a healthy balanced diet [17,18], such as the Mediterranean diet [17].

A body of research has focused on the role of individual whole foods rich in nutrients hypothesised to be beneficial for muscle (myoprotective), such as protein-rich whole foods, to establish the role of protein source (quality), quantity, and timing of intake in muscle ageing [14,15]. Others have evaluated the influence of selected foods in the context of whole diet to understand their individual contribution to muscle health and function [17,18], and the potential cumulative and synergistic benefits of nutrients and non-nutrients in these foods for muscle [16]. To our knowledge, a systematic evaluation of the studies that used a whole food approach in relation to muscle health and sarcopenia in older adults is lacking. We have recently found limited evidence for the role of liquid milk in muscle health in older adults, that has potential to have beneficial effects, acting through anabolic, anti-oxidative, anti-inflammatory, and immunomodulating pathways implicated in sarcopenia [16]. To examine further whether other whole foods rich in specific nutrients influence muscle health, we aimed to summarise evidence from observational and intervention studies that used meat, fish, eggs, fruit and vegetables, and non-liquid dairy (yoghurt and cheese) in relation to muscle mass, function, and sarcopenia in older adults aged 50 and over.

## 2. Materials and Methods

### 2.1. Protocol and Registration

This study was performed in accordance with the Preferred Reporting Items for Systematic Review and Meta-Analyses (PRISMA) guidelines [20]. All procedures were agreed beforehand and were registered on the International prospective register of systematic reviews (PROSPERO; http://www.crd.york.ac.uk/prospero) on 29 October 2018 as CRD42018114406.

### 2.2. Eligibility Criteria

This review included both observational studies (cross-sectional and longitudinal) and intervention studies (randomised control trials (RCTs)) published in English in the last 20 years (from the year 2000 until 5 March 2020), to capture the period when the nutrition investigations about the role of diet in age-related health outcomes have gone beyond a single-nutrient to a whole food approach. To be included in the review, studies were required to have a sample size of at least 50 participants for observational studies, or 10 participants per group for RCTs. We included all studies that clearly described exposure (i.e., whole foods: type, amount/dosage, and frequency/duration of consumption) and assessed at least one of the following outcome measures related to muscle health: muscle strength, mass, function, and sarcopenia. All outcome measures related to skeletal muscle protein metabolism were not considered and were beyond the scope of this review. For RCTs, we considered studies with intervention duration of at least 6 weeks (a whole food with or without an exercise component), which has been evaluated as a minimum timeframe used in the studies investigating the relationship between liquid milk and muscle-related outcomes in our recent review [16]. We excluded studies that used biomarkers of macro and micronutrients status instead of whole foods as a measure of dietary exposure, and those examining liquid foods only (e.g., juices, liquid milk without other dairy products, and liquid protein-based supplements) or reconstituted liquid/semisolid foods from powder or fortified/enriched whole foods in relation to muscle health. Observational studies that used diet/dietary pattens as exposures from which the individual effect of whole foods could not be estimated, were excluded. We also excluded reviews, trial protocols, conference abstracts, book chapters, studies with no control/placebo group, and case reports for RCTs, opinions or commentaries, and mixed methods research articles for observational studies. We included studies of adults (both sexes) aged ≥ 50 years (to maximise the number of individuals who may experience age-related decline in muscle mass and function), from all races/ethnicities, all settings (community, care homes, and hospitals) around the world. A complete list of inclusion and exclusion criteria is presented in Table S1.

### 2.3. Information Sources

Electronic searching of four databases (MEDLINE, Embase, Web of Science, and Cochrane Central Register of Controlled Trials) was performed from the year 2000 up to 5 March 2020. Reference lists from retrieved studies and previously published systematic reviews were also examined for potentially eligible papers.

### 2.4. Search Strategy

Specific search terms were applied to type of whole food, outcomes related to muscle mass, function and sarcopenia, and age. Whole food was defined as meats (beef, pork, lamb, poultry, and processed meats), fish, eggs, fruit and vegetables (FV), and solid/semisolid milk (yoghurt and cheese). We included only commonly consumed whole foods that could be purchased in food stores and markets, and judged to be accessible to older adults as a part of balanced diet. For muscle function and sarcopenia, we included muscle mass, muscle strength, walking speed, appendicular lean mass (ALM), skeletal muscle index, and Timed Up-and-Go (TUG) test. Our search combined both MeSH and free-text terms related to whole foods and sarcopenia. The search results were exported into a Microsoft Excel sheet and duplicates removed.

This is an example of our search strategy in MEDLINE: ((exp fish/or exp eggs/or exp meat/or exp red meat/or exp fruit/or exp fruit vegetable juices/or fruit.tw exp vegetables/or exp yoghurt/or exp cheese/) OR (“semisolid milk” OR “yoghurt” OR “cheese” OR “vegetable” OR “fruit” OR “meat” OR “eggs” OR “fish”).tw) AND ((exp Muscle Weakness/or exp Muscle, Skeletal/or exp Aged/or exp sarcopenia/or exp “Aged, 80 and over”/) OR (“muscle mass” OR “muscle strength” OR “grip strength” OR “walking speed” OR “gait speed” OR “appendicular lean mass” OR “skeletal muscle index” OR “physical performance” OR “Timed Up-and-Go test” OR “muscle wasting” OR “age-related muscle loss” OR “myopenia” OR “dynapenia” OR “sarcopenia” OR “sarcopenic”).tw) AND ((exp aged 45 and over/ OR exp Aged/OR exp Aged, 80 and over/) OR (“aged 50 and over” OR “adults aged 50 and over” OR “older adults” OR “elderly”).tw.). An example of the initial search strategy and terms for two databases is presented in Table S2.

### 2.5. Study Selection

**Figure 1:**
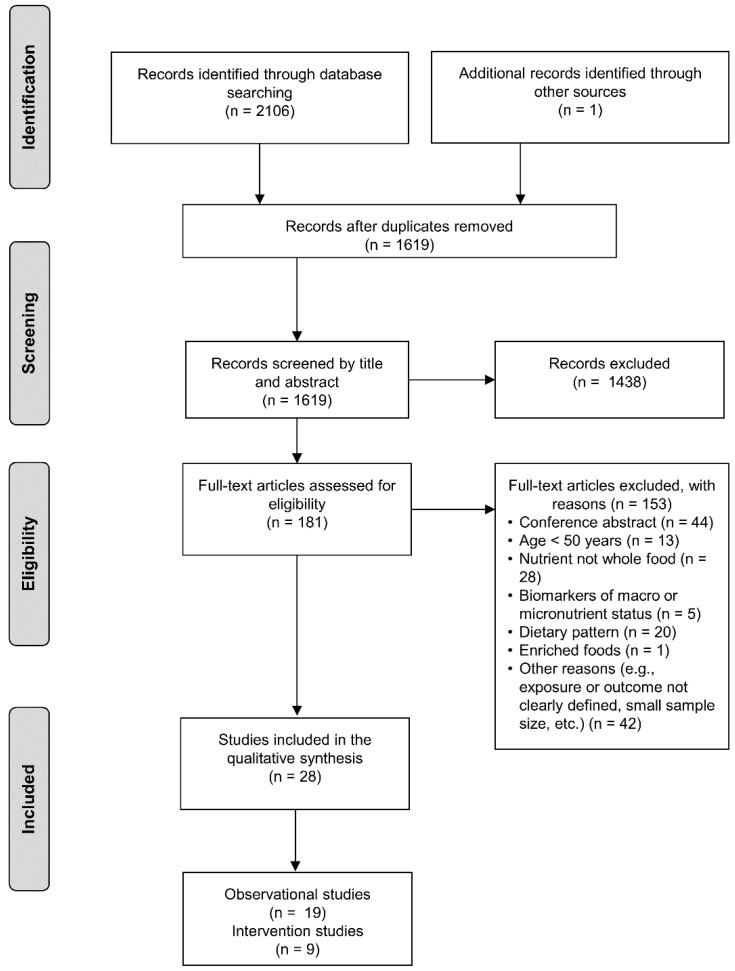
PRISMA flow diagram of the selected studies. PRISMA, Preferred Reporting Items for Systematic Reviews and Meta-Analyses.

To identify relevant studies, all records were screened independently for eligibility by two authors (CH and LD) with any disagreements resolved by a third reviewer (AG). Firstly, title and abstracts were screened using our pre-determined inclusion and exclusion criteria, and the studies that were not relevant were removed. Following this, full-text papers were evaluated using the same inclusion and exclusion criteria and a modified version of a PRISMA flow diagram of the study selection process is presented in Figure 1.

### 2.6. Data Extraction and Data Items

Two reviewers extracted data (CH, LD), and the third reviewer (AG) checked it for accuracy and synthesized the results. Relevant data from observational studies (Table 1) and RCTs (Table 2) was extracted including participants details (mean age or age range, sex, setting, and population), whole food (type, amount, frequency, and duration of intervention/exposure), training (if applicable: type, duration, frequency, and amount/dosage), and control group. Primary and secondary outcome measures for all studies (e.g., sarcopenia, skeletal muscle mass (ALM, skeletal muscle mass index), muscle strength (grip strength (GS), leg extension strength), walking speed (gait speed), and physical performance (TUG test, Senior Fitness Test (SFT)) with intervention/exposure effect were described in Table 1 and Table 2.

### 2.7. Risk of Bias in Individual Studies

Risk of bias was assessed by two researchers (CH and LD) with any discrepancies resolved via discussion. The risk of bias of observational studies was assessed using the Newcastle–Ottawa Scale (NOS) and the NOS manual (http://www.ohri.ca/programs/clinical_epidemiology/oxford.asp) (Table S3). The quality of RCTs was assessed using the revised version of the Cochrane Risk of Bias Tool (RoB 2) [49]. Briefly, we assessed five specific domains as ‘high risk of bias’, ‘low risk of bias’, or ‘some concerns’ to establish the overall risk of bias: (i) bias arising from the randomisation process; (ii) bias due to deviation from intended interventions; (iii) bias due to missing outcome data; (iv) bias in measurement of the outcome, and (v) bias in selection of reported results (Figure S1). Evidence quality (risk of bias) and quantity (number of studies with the statistically significant associations/effects) were used to evaluate the strength of evidence (e.g., strong, moderate, weak, inconclusive, and limited) for each whole food group in Table 3.

### 2.8. Summary Measures and Qualitative Synthesis of Results

Because of the relatively small number of studies and their methodological differences, the summary of the study findings was evaluated descriptively. Where applicable, we described associations between intake of whole food and muscle health outcomes versus those not exposed (Table 1), and odds ratios (OR) and hazard ratios (HR) of sarcopenia, and changes in muscle mass, strength and function in an intervention group (a whole food or whole food + exercise) versus control group (Table 2).

## 3. Results

### 3.1. Study Selection

Figure 1 describes the flow of the studies selected for the review. A total of 2106 articles were identified after the electronic searches with one additional article identified through hand searches. After removal of 488 duplicate records, 1619 articles were screened by title and abstract, and 1438 were excluded as not relevant. A total of 181 full-text articles were screened further for eligibility based on inclusion and exclusion criteria, and 152 were removed for the following reasons: (i) 44 were conference abstracts; (ii) 13 included participants <50 years old; (iii) 28 investigated nutrients; (iv) 5 used serum biomarkers to determine exposure; (v) 20 investigated dietary patters; (iv) 1 used enriched whole foods, and (vii) 44 were excluded for other reasons (e.g., either exposure or outcome not clearly defined; non-relevant outcome; small sample size; etc.). A total of 28 studies were included in the qualitative synthesis.

#### 3.1.1. Observational Studies

Nineteen observational studies examined the association between whole foods, muscle-related outcomes and sarcopenia in older adults (aged ≥ 50 years). Of these, three studies examined the relationship between meat intake (red meats, poultry, and processed meats) and muscle mass and function [21,22,23]; five examined the intake of fruit and vegetables in relation to sarcopenia and muscle function [24,25,26,27,28], and eight studies investigated the link between multiple whole foods and various muscle-related outcomes [29,30,31,32,33,34,35,36]. The individual whole foods were: fatty fish, white fish and shellfish, legumes, soy, nuts and seeds, eggs, and the components of the Nordic Dietary Score (NDS), including Nordic cereal (rye, oats, barley) [29,30,31,32,33,34,35,36]. Three studies investigated the association between dairy intake (including yogurt and cheeses) and muscle mass and function [37,38,39]. For the latter, only studies that included semi-solid and solid dairy (i.e., non-liquid dairy with or without liquid milk) as individual food items in relation to muscle health were included in the review (Table 1). Studies with liquid milk as a whole food in relation to muscle health have been evaluated previously [16].

To assess diet, ten studies used food frequency questionnaires (FFQ) [24,27,29,30,31,33,34,35,37,38], four studies employed diet histories [21,23,25,39], one used 3-day diet record [36], one 24-h dietary recall [22], two used lifestyle questionnaire [26,32], and one study used a single question for a specific food group [28]. Muscle-related outcomes varied across the studies; six examined muscle mass (total, lean, appendicular, skeletal muscle index, etc.) [21,22,31,36,37,38], nine examined muscle function (functional tasks, Short Physical performance Battery (SPPB), gait speed, TUG, chair rise, balance, Senior Fitness Tet, etc.) [23,25,26,27,30,33,36,37,39], seven investigated muscle strength (grip strength, knee extension strength) [21,26,29,32,35,37,39], and only three studies examined the relationship between sarcopenia and whole foods [24,28,34].

#### 3.1.2. Intervention Studies

Nine intervention studies met the inclusion and exclusion criteria [40,41,42,43,44,45,46,47,48]. All except one [41] were RCTs of which four included resistance exercise with nutrition intervention [40,42,44,45]. Two studies used the same nutrition intervention protocol with older adults with [46] and without sarcopenia [47], and two articles were secondary analyses [42,45] of the primary studies (RCTs) [40,44] reporting the effect of the intervention on different muscle-related outcomes. Whole foods used in the nutrition interventions were meats (lean red meats (beef, veal, lamb), pork, and chicken) [40,41,42], fruit and vegetables [43], multiple whole foods (beef or soy-based products) added to a lacto-ovo vegetarian (LOV) diet [44,45], ricotta cheese [46,47], and eggs [48]. Number of participants, participants’ gender, intervention duration, and muscle-related outcomes varied across the studies. The number of participants included in interventions ranged from 21 to 100, and all except two studies [40,44] (and their secondary analyses [42,45]) included men and women. Whole food interventions lasted from 12 weeks (five studies [41,44,45,47,48] and 16 weeks (one study [43]), through to 3 months (one study [46]) and 4 months (one study [40]). Six studies examined the effect of an intervention on different measures of muscle mass [40,42,44,46,47,48], muscle function (five studies [40,41,43,45,47]), and muscle strength (six studies [41,42,43,45,46,47]).

### 3.2. Risk of Bias within the Studies

#### 3.2.1. Risk of Bias in Observational Studies

Table S3 shows the risk of bias scores for each study across nine criteria (e.g., study design, participants, diet quality (assessment), outcome measurements, analysis), and the overall quality rating (risk of bias score) based on the NOS tool. Ten studies (53%) [21,22,23,27,28,31,34,35,37,39] had low risk of bias (score range +4 to +8), and eight studies (42%) [24,25,29,30,32,33,36,38] had medium risk of bias (score range 0 to +3). One study had high risk of bias (score −4) [26]. Per exposure (whole foods), three studies with meat had low risk of bias [21,22,23]; five studies with fruit and vegetables had medium (three studies) [24,25,28], low (two study) [27,28], or high (one study) [26] risk of bias; eight studies with multiple whole foods had medium (five studies) [29,30,32,33,36] or low (three studies) [31,34,35] risk of bias; and three studies with semi-solid dairy/cheeses had low (two studies) [37,39] or medium (one study) risk of bias [38] (Table S3).

#### 3.2.2. Risk of Bias in Intervention Studies

Figure S1 shows the risk of bias for included intervention studies according to the Cochrane Collaboration tool for risk of bias assessment [49]. In all, three studies were judged to have low risk of bias [40,42,47], three had some concerns regarding bias [43,46,48], and three had high risk of bias [41,44,45]. The high risk was mainly attributed to three domains (randomisation, deviations from intended intervention, and missing outcome data). Per exposure (whole foods), of three studies with meat intervention one had high risk of bias; one study with fruit and vegetables intervention had some risk of bias; two studies with multiple foods had high risk of bias; two studies with semi-solid dairy had low and some risk of bias; and one study with eggs intervention had some concerns for bias.

### 3.3. Characteristics and Results of Individual Studies

#### 3.3.1. Characteristics and Results of Observational Studies by Whole Foods

##### Meats

Two cross-sectional studies looked at beef consumption and muscle mass [21,22]. In a study of 142 community-dwelling older adults (aged 60–88 years), Asp et al. (2012) [21] determined the relationship of beef consumption with nutrition status, body composition, muscle strength (GS), and biochemical measures of vitamin and mineral status, blood lipids, and inflammation. Dietary intake was assessed during the past 12 months using the Diet History Questionnaire, and blood samples were collected. GS and anthropometry were measured, including BMI, mid-arm and calf circumference, and triceps skinfold (TSF). Mid-arm muscle area was calculated and sagittal abdominal diameter (SAD), a measure of intra-abdominal fat was measured using a Holtain-Kahn Abdominal Caliper. Beef intake was positively associated with mid-arm area (R = 0.128, *p* = 0.03); however, no correlations were found between beef consumption and calf circumference, SAD or TSF. GS was not correlated with beef intake. A 1 ounce/day (~28 g/day) increase in beef consumption predicted a 2.3 cm^2^ increase in mid-arm muscle area. The authors concluded that lean cuts of beef in moderation may be a healthy way to increase protein consumption for muscle health in older adults.

Morris and Jacques (2013) [22] sought to clarify the role of leisure-time activity and protein quality in the preservation of skeletal muscle during ageing, and how such influences interact in 2425 community-dwelling American older adults aged ≥ 50 years in the National Health and Nutrition Examination Survey (NHANES 2003–2006). Total beef intake was estimated using two 24-h diet recalls, and body composition was measured using dual-energy X-ray absorptiometry (DXA), and appendicular skeletal muscle mass (ASM) index from anthropometric measures. In non-obese subjects (n = 1789), the mean ASM index was modestly, but significantly increased in association with higher usual beef intakes. Dietary intake of protein or beef appeared to be more important to non-obese subjects who performed muscle-strengthening or vigorous activity exercises. Each 100 g increase/week in beef intake was associated with a 0.10 (*p* = 0.04) and 0.13 (*p* = 0.006) increase in ASM index in non-obese participants performing either vigorous or muscle-strengthening exercises, respectively. In obese participants (n = 636), beef intake was positively associated with the ASM index only in those performing vigorous aerobic activities (β = 0.12, *p* = 0.18).

In the Seniors-ENRICA cohort study (Study of Nutrition and Cardiovascular Risk Factors in Spain) including 2982 Spanish older adults (aged ≥ 60 years), higher consumption of processed meat at baseline (2008–2010) was associated with poorer physical functioning over a 5.2-year median follow-up [23]. Meat intake was assessed by a validated computer-assisted face-to-face diet history, and functional tasks (agility and mobility) by the Roscow–Breslau scale and Short Physical Performance Battery (SPPB). Participants in the highest tertile of processed meat had a higher risk of impaired agility (HR = 1.33; 95% CI 1.08–1.64, *p* = 0.01), with a 100 g/day increase in processed meat consumption resulting in a 23% higher risk. Higher processed meat consumption was also associated with the increased risk of lower extremity function (SPPB) (HR = 1.32; 95% CI 1.02–1.68, *p* = 0.04). No associations between red meats of any kind and poultry and muscle function were found.

In summary, higher beef intake as a source of protein and other nutrients was positively associated with muscle mass, especially in older adults performing vigorous exercise, whilst higher intake of processed meat increased the risk of poor physical functioning over time.

##### Fruit and Vegetables (FV)

Two cross-sectional [24,28] and three prospective studies [25,26,27] investigated the link between FV intake and muscle-related outcomes in older adults, and all reported beneficial effects. In the Fourth Korea National Health and Nutrition Examination Survey (KHNES; 2008–2009) of 823 men and 1089 women aged ≥ 65 years, higher FV intake was associated with a lower risk of sarcopenia. Sarcopenia was defined as low height- and fat-mass adjusted lean mass, and FV intake were assessed by food frequency questionnaire (FFQ). Men in the highest quintile of vegetables, fruit and total FV intake had a lower risk of sarcopenia (OR = 0.48, 95% CI: 0.24–0.95; OR = 0.30; 95% CI: 0.13–0.70; OR = 0.32; 95% CI: 0.16–0.67, respectively) compared to those in the lowest quintile. However, only the highest quintile of fruit intake was associated with a lower risk of sarcopenia in women (OR = 0.39; 95% CI: 0.18–0.83), compared to those in the lowest quintile [24].

In the Study on Global Ageing and Adults Health of 14,585 older adults aged ≥ 65 years from low- and middle-income countries (China, Ghana, India, Mexico, Russia, and South Africa), increased fruit but not vegetable consumption was associated with lower prevalence of sarcopenia [28]. Participants were asked how many servings of FV they ate on a typical day (categorised in quintiles, Q1–Q5), whilst sarcopenia was defined as the presence of low skeletal muscle index (SMI) based on population formula in combination with either low GS or gait speed. In unadjusted analysis, higher fruit consumption was associated with lower prevalence of sarcopenia in women (21% in Q1 (0 servings) versus 7.9% in Q5 (≥4 servings)). In adjusted analysis, Q5 of fruit was associated with lower odds of sarcopenia (OR = 0.60, 95% CI 0.42–0.84, *p* < 0.01), compared to Q1 in all participants, and in women (OR = 0.42, 95% CI 0.24–0.73, *p* < 0.01), but not in men. No associations were found for vegetable intake [28].

Three studies examined the change in muscle-related outcomes over time in relation to FV intake [25,26,27]. García-Esquinas et al. (2016) [25] sought to examine the dose-response association between FV consumption and the risk of physical frailty in three independent cohorts of community-dwelling older adults (n = 2926). Frailty was measured using a slight modification of the phenotypic criteria proposed by Fried et al. (2001) [50]. FV consumption was obtained using a validated computerised diet history or FFQ. Higher consumption of FV was associated with lower risk of incident frailty, and better walking speed. Specifically, an inverse dose-response relation was found between baseline consumption of FV and the risk of slow walking speed. The ORs for participants who consumed 1, 2 or ≥3 portions of fruit/day (1 portion = 120 g) to those with no consumption were, 0.59 (95% CI: 0.27–0.90), 0.58 (95% CI: 0.29–0.86), and 0.48 (95% CI: 0.20–0.75), with a *p* < 0.04, respectively. The corresponding values for vegetables (1 portion = 150 g) were 0.69 (95% CI: 0.42–0.97), 0.56 (95% CI: 0.35–0.77), and 0.52 (95% CI: 0.13–0.92), with a *p* < 0.01. When FV were analysed together, the pooled ORs of incident physical frailty were 0.41 (95% CI: 0.21–0.60), 0.47 (95% CI: 0.25–0.68), 0.36 (95% CI: 0.18–0.53), and 0.31 (95% CI: 0.13–0.48), with a *p* < 0.01 for participants who consumed 2, 3, 4, or ≥5 portions/day, respectively, compared to those who consumed ≤1 portion/day [25].

In the African American Healthy Study of 432 older adults (mean age 59.2 ± 4.4 years) from St. Louis, MO, USA, FV intake was assessed using questions from the Behavioral Risk Factor Surveillance System (BRFSS) survey, and the outcome measures included gait speed, GS, Lower Body Function Limitation (LBFL; 5 activities), and SPPB. Longitudinally, higher intakes of vegetables other than carrots, salads, or potatoes were associated with better GS (β (SE) = 0.11 (0.04), *p* = 0.01), while fruit juice was associated with worse changes in GS over time [26].

The Perth Longitudinal Study of Aging in Women, Australia examined the associations of FV and muscle strength (GS), physical performance (TUG), and falls-related hospitalisation in 1429 postmenopausal women (aged ≥ 70 years) [27]. Diet was assessed using an FFQ validated by the Cancer Council of Victoria. Higher total vegetable intake was associated with lower odds for weak GS and slow TUG. An increase in vegetable serving intake (75 g per serving) reduced the odds of weak GS (<22 kg) by 13% (*p* = 0.01). Compared with low vegetable intake (<2 servings/day), high vegetable intake (≥3 servings/day) was associated with 31% lower odds of weak GS in both the age and multivariate-adjusted models (*p* = 0.02). An inverse association was also demonstrated between vegetable intake and TUG: 12% lower odds of slow TUG for every 75 g/day increase in vegetable intake. Compared with low vegetable intake (<2 servings/day), high vegetable intake (≥3 servings/day) was associated with 31% lower odds of slow TUG. Fruit intake (per 150 g serving) was inversely associated with GS in both the age and multivariate adjusted models. High (≥2 servings/day) in comparison with low (<1 serving/day) fruit intake was associated with 30% lower odds of weak GS, only in the age adjusted model [27].

In summary, higher intake of fruit was associated with 40%–60% lower odds of sarcopenia, especially in older women [24,28]. Having ≥3 portion/day of FV (1 fruit portion = 120 g; 1 vegetable portion = 150 g) was associated with 50% reduced odds of developing slow walking speed [25] in older adults, whilst consuming ≥3 servings/day of vegetables (1 serving = 75 g) was associated with 31% reduced odds of weak muscle strength (GS) and poor physical performance (TUG) in older women [27].

##### Multiple Whole Foods

Eight observational studies [29,30,31,32,33,34,35,36] (four cross-sectional [29,30,31,34], one cross-sectional and prospective [32], and three prospective studies [33,35,36]) examined the relationship between multiple whole foods and muscle-related outcomes either as a part of dietary pattern (index) or as an independent exposure.

In the cross-sectional studies, significant associations were reported with the following foods. Robinson et al. (2008) examined the relationship between diet and GS in older men and women (n = 2983) aged 59 to 73 in the Hertfordshire Cohort Study, UK [29]. Diet was assessed using an FFQ and GS was measured. In men, higher consumption of fruit, fatty fish, and breakfast cereals but lower meat consumption (including red and white meats) was associated with higher GS. In women, higher GS was associated with higher consumption of fruit and fatty fish, but there was no association with breakfast cereals or meat consumption. The most important association between diet and GS was with fatty fish consumption. Specifically, each additional portion of fatty fish consumed per week was associated with a gain in GS of 0.43 kg (*p* = 0.005) in men and 0.48 kg (*p* < 0.001) in women in analyses adjusted for height, age, and birth weight [29].

Using 628 participants aged 63–73 years from the same cohort, Martin et al. (2011) examined the association between several whole foods and physical performance, measured using the SPPB (i.e., the time taken to complete a 3-m walk, chair-rise test, and one-legged balance test) [30]. Foods included fruit and vegetables, nuts, meat and meat dishes, white fish and shellfish, and oily fish assessed using a validated FFQ. In women, an inverse association between vegetables (*p* = 0.02), white and shellfish (*p* = 0.04), and oily fish (*p* = 0.007) and 3-m gait speed was observed after multivariable adjustments. Furthermore, higher nuts (*p* = 0.01) and vegetables intake (*p* = 0.02) was associated with better char-rise time in women. However, only associations with vegetables remained robust after adjustments for the effect of confounders. No association between whole foods and physical performance were observed among men [30].

In the Fourth and Fifth KHANES (2008–2011) study of 3285 Korean older adults aged ≥ 65 years, food groups consisting of meat, fish, eggs, legumes, and fruit and vegetables were analysed in association with appendicular skeletal muscle mass (ASM) measured by DXA [31]. Women who consumed the recommended levels of vegetables (≥5/day from a list of 12 vegetables: Chinese cabbage, radish, dried radish leaves, bean sprouts, spinach, cucumber, hot peppers, carrots, pumpkin, cabbage, tomatoes, and mushrooms) has significantly lower likelihood of low ASM (OR = 0.52, 95% CI: 0.30–0.89) than those not meeting the recommendations. No associations were observed with other food groups, and no associations between any of the food groups and ASM were observed in men [31].

A study of 834 community-dwelling Chinese older adults (aged between 60–92 years) investigated the association between sarcopenia and lifestyle, including diet. Diet was measured using a validated FFQ consisting of grains/cereals, fruit and vegetables, eggs, fish/shrimp, nuts, meat (pork, beef, mutton, and poultry), milk/milk products, and legumes [34]. Sarcopenia was determined based on the Asian Working Group for Sarcopenia definition [51]. Of all foods, only higher nuts consumption was associated with reduced risk of sarcopenia. Specifically, women with sarcopenia had significantly lower frequency per week of nut consumption than those without sarcopenia (mean (SD): 0.05 (0.22) versus 0.81 (2.11), *p* = 0.02). In the multivariable model, frequency per week of nuts consumption were significantly associated with reduced odds of sarcopenia (OR = 0.72, 95% CI 0.53–0.99, *p* < 0.05). No significant associations between sarcopenia and any food groups were observed in men [34].

Kojima et al. (2015) used a combination of cross-sectional and prospective analyses to investigate which modifiable lifestyle factors have beneficial or detrimental effects on the age-related decline in isometric knee extension strength (KES in Newtons, N) among 575 Japanese community-dwelling women aged between 75–85 years [32]. Frequencies of intake of ten whole foods habitually consumed in Japanese diet consisting of green and yellow vegetables, potatoes, fruit, soy products, seaweeds, seafood, meat, egg, milk, and oils and fats were assessed by a close-ended lifestyle questionnaire at baseline (2008). Cross-sectionally, there were no significant associations between KES and the frequency of intake any food groups. However, prospective analyses revealed that daily intake of soy products and green or yellow vegetables at baseline was protective of KES decline over 4 years. The decrease of KES in participants who ate soy products almost every day (17.87 N) was approximately 69% of that in those who ate soy products once per 2 days or less (26.06 N, *p* = 0.03), and the decrease of KES in participants who ate green or yellow vegetables almost every day (18.82 N) was approximately 60% of that in those who ate vegetables once per 2 days or less (31.46 N, *p* = 0.02). In contrast, women who ate seafood almost daily had significantly greater decrease (1.5 times) in KES (24.68 N) compared with those who ate seafood once per 2 days or less (16.88 N, *p* = 0.02), explained by potentially negative effects of methyl mercury in fish on muscle strength, and low power in data [32].

Three studies looked at the prospective association between whole foods included in a dietary pattern [33,35] or selected as individual exposure [36] and muscle mass and function in the community-dwelling older adults. Two prospective investigations were conducted in the Helsinki Birth Cohort Study (1072 older adults born 1934–1944), Finland, using the elements of the Nordic Diet Score (NDS). The NDS included Nordic fruit and berries (apples, pears, and berries), Nordic vegetables (tomatoes, cucumber, leafy vegetables, roots, cabbages, and peas), Nordic cereals (rye, oats, and barley), Nordic fish (salmon and freshwater fishes), red and processed meat, assessed with a validated FFQ at mean age of 61 years (2001–2004). The score and individual foods were examined in relation to change in the Senior Fitness Test (SFT) [33], GS, leg strength (knee extension), and muscle mass at age of 71 years (2011–2013) [35]. In women, high consumption of fruit and berries (*p* = 0.01), and cereals (*p* = 0.03) were positively related to the overall SFT score, whereas consumption of red and processed meat were inversely associated with the SFT score (*p* = 0.001). In men, high consumption of cereals (*p* = 0.04) was significantly associated with better overall SFT score [33]. Furthermore, Nordic cereals intakes were positively related to leg strength change (*p* = 0.05), whilst red and processed meats were inversely related to GS change (*p* = 0.001) only in women [35].

In the Framingham Offspring Study, Boston, MA, USA of 1016 men and 1333 women (median age 52 years at baseline), Bradlee et al. (2018) examined the effects of the primary food sources of animal protein (red meat, poultry, fish, and dairy) as well as plant protein (legumes, nuts, sees, and soy), alone and in combination with physical activity on longitudinal changes in skeletal muscle mass (SMM) and functional decline over time [36]. Diet was assessed from 3-day dietary records, SMM was measured using bioelectrical impedance (BIA) (a 9-year follow-up), and functional status was assessed using the Roscow–Breslau scale and the Nagi scale (a 13-year follow-up). Higher intakes of protein-source foods including red meat, poultry, fish, dairy, and soy, nuts, seeds, and legumes were associated with higher percentage of SMM over 9 years, especially among women. Specifically, women who consumed ≥2 servings of red meats (beef, lamb, and pork)/day had an extra mean 1.2% SMM (*p* < 0.001) compared with those consuming <0.85 servings/day (1 serving = 1 ounce (28.35 g), cooked). In all participants, those who consumed ≥3 servings/day of poultry or fish had an extra mean % SMM of 0.8 (*p* = 0.02) and 1.2 (*p* =0.001), respectively, compared with those consuming <1 serving a day (1 serving = 1 ounce (28.35 g), cooked). A non-significant 20% reduction in developing functional impairment in ≥2 tasks was observed in participants consuming ≥1 serving/day of dairy (1 serving = 1 cup of milk or yoghurt, 1–1.5 ounce cheese) or ≥1 (women) or ≥2 (men) servings of poultry and fish compared with those consuming less [36].

In summary, the following multiple whole foods were associated with muscle mass and function either cross-sectionally or over time, especially in women. In cross-sectional associations, higher intake of fatty fish was beneficial for muscle strength (GS) in both men and women [29], whilst total fish (white/shell/fatty) and vegetables intake were beneficial for muscle function (gait speed and chair rises, respectively) only in women [30]. Consuming recommended levels of vegetables a day (≥5 servings/day) was associated with higher muscle mass [31], and more frequent nuts consumption per week reduced the odds of sarcopenia by 30% only in women [34]. In longitudinal associations with all participants, daily intake of soy products, green or yellow vegetables was associated with lower decline in muscle strength (knee extension strength) [32], whilst intake of ≥3 servings/day (≥85.1 g/day) of poultry or fish was associated with 0.8%–1.2% higher muscle mass [36]. However, consuming ≥2 servings (56.7 g) a day of red meat (beef, lamb, and pork) was beneficial for muscle mass in women [36], whilst higher intake of red with processed meat was associated with worse change in muscle strength (GS) in women [35]. Furthermore, higher intake of Nordic fruit/berries and cereal were associated with lower decline in muscle strength (leg strength) only in women [35].

##### Dairy (including Semi-Solids and Cheese)

Two cross-sectional [37,38] and one prospective study [39] investigated the association between dairy consumption (including yogurt and cheese) and muscle mass, strength, and function.

Radavelli-Bagatini et al. (2013) evaluated the association between dairy intake, lean, and fat mass and physical function in 1456 community-dwelling women aged between 70 and 85 years who participated in the Calcium Intake Fracture Outcome Study (CAIFOS), Western Australia [37]. A validated FFQ was used to assess dietary intake including dairy products (milk, yogurt, and cheese) consumption in the previous 12 months at study entry (1998). Body composition was measured with DXA, and muscle function evaluated by GS and TUG. Women in the second and third tertiles (≥2.2 servings/day) of dairy intake had significantly greater whole-body lean mass and ASM compared with those in the first tertile (≤1.5 serving/day). There were no significant differences between the three tertiles in whole body fat mass. GS was greater in those in the third tertile of dairy intake compared with those in the first tertile. Women in the third tertile compared to the first, had lower odds for slow TUG test performance (OR = 0.77, 95% CI: 0.59–1.00, *p* = 0.04) [37]. The authors repeated the analyses in 564 women aged 80 to 92 years at 10-year follow-up [38]. Higher dairy intake was associated with greater muscle mass, independent of a range of covariates (age, body size, energy intake, physical activity level, smoking habit, alcohol consumption, and use of calcium or vitamin D supplementation). Women in the highest tertile (≥2.2 servings/day) had had a 4.0% higher ASM (*p* = 0.04) compared with the lowest tertile (≤1.5 serving/day), which remained significant after multivariate adjustments (3.3%, *p* = 0.01). One serving of dairy was equivalent to 200 g of yogurt, or 40 g hard, firm soft and low-fat cheese, or 120 g for cottage and ricotta cheese [37,38].

In the prospective study of 1871 older adults aged ≥ 60 years participating in the Seniors-ENRICA cohort, Spain habitual consumption of dairy products was evaluated in relation to the risk of frailty, and muscle function (GS, walking speed) [39]. The study used computerised diet history to assess consumption of up to 880 foods during the previous year. One serving of yogurt was 125 mL, and cheese was 40 g. Greater consumption of low-fat dairy products, low-fat milk in particular, was associated with lower risk of frailty mostly due to a lower risk of slow walking speed. Consuming ≥7 servings per week of dairy was associated with the reduced risk of slow walking speed (OR = 0.64, 95% CI: 0.44–0.92, *p* = 0.01). There were no associations with GS [39].

In summary, consuming ≥2 servings of dairy products (including yogurt and cheese) was associated with better lean muscle mass, and muscle function (GS and TUG) in older women (aged ≥ 70 years) [37,38]. Consumption of ≥7 servings of dairy a week (e.g., 125 mL of yogurt or 40 g of cheese a day) was associated with a reduced risk of slow walking speed in older adults [39].

#### 3.3.2. Characteristics and Results of Intervention Studies by Whole Foods

##### Meats

In a 4-month RCT with 100 older women (aged 60–90 years) residing in 15 retirement villages in Melbourne, Australia, Daly et al. (2014) examined the effect of protein-enriched diet (beef, veal, or lamb) in combination with a progressive resistance training (PRT) on lean muscle tissue mass (LTM), muscle size, muscle strength and function, and circulating inflammatory biomarkers (IL-6), blood lipids, and blood pressure [40]. Women were allocated to the intervention group (PRT twice weekly + 160 g of cooked lean red meat (~1.3 g of protein/kg body weight (BW) a day divided into two servings for 6 days a week) and control group (PRT twice weekly + ≥1 serving (~75 g) of pasta or rice a day (~25–35 g of carbohydrates)). Both groups received 1000 IU of vitamin D_3_ a day over the study period. Throughout the study, protein intake was greater in the intervention group compared with control group (1.3 ± 0.3 versus 1.1 ± 0.3 g/kg BW/day (*p* < 0.05)), and carbohydrates intake was ~20–40 g greater in controls. The intervention group had a 0.5 kg (95% CI: 0.1–0.8 kg) greater gains in total body LTM (*p* = 0.007), attributed to a greater increase in leg LTM (intervention group: 0.3k g 95% CI: 0.2–0.5 kg; control: 0.1 kg 95% CI: −0.04–0.3kg). The intervention group experienced a significant 0.5 kg (95% CI: −0.9–0.03kg) decrease in fat mass compared with controls (*p* < 0.05). After 4-month intervention, the intervention group had, on average, 18% (3%–34%) greater increase in leg extension muscle strength than controls (*p* = 0.01). Both groups experienced PRT-induced improvements in muscle function assessed by TUG, 4-square step test, and 30 s sit-to-stand test (e.g., 4-month change in TUG performance of −14.4 s (95% CI: −19.5–−0.1) in the intervention versus −16.4 s (95% CI: −29.7–−0.1) in control group). IL-6 decreased significantly (*p* < 0.05) in the intervention group, but no between-group differences were observed for blood lipids and blood pressure over the study period. The authors concluded that a protein enriched diet corresponding to ~1.3 g/kg BW/day achieved through lean red meat was a safe nutritional intervention in enhancing the effect of PRT on LTM and muscle strength, and reducing inflammation in older women. The follow-up analysis of RCT data [42] examined the effect of the PRT + lean red meat intervention on health-related quality of life (HR-QoL) assessed by the SF-36 Health Survey questionnaire, in relation to lower limbs maximum muscle strength, and LTM. The study observed a significant net benefit of the intervention on the overall HR-QoL (*p* = 0.009), mainly due to an increased physical component summary score (PCS) (*p* = 0.007), and no significant change in the mental component score (MCS). Significantly improved PCS sub-domains in the intervention group included physical functioning (*p* = 0.04), physical functioning role limitation because of physical health (*p* = 0.04), and physical pain (*p* = 0.02). Furthermore, changes in leg muscle strength, but not leg LTM, were positively associated with changes in overall H-QoL (β = 2.2, 95% CI: 0.1–4.3, *p* = 0.04). No association between the muscle strength or LTM change with the change in PCS was observed, but the MCS change was positively associated with leg muscle strength (*p* = 0.008). In summary, the PRT + lean red meat intervention was associated with a modest but significant 7% increase in health-related quality of life in older women, which was contributed by physical and not mental health components of the SF-36 questionnaire. Intervention-induced changes in lower limb strength but not in lean tissue mass was associated with overall HR-QoL and mental health [42].

In a pilot quasi-experimental study, Charlton et al. (2016) investigated the effect of pork intake four times a week over 12 weeks on body mass, strength and cognitive function compared to chicken diet in 48 community-dwelling older adults aged 65 to 89 years living in the retirement villages in North South Wales, Australia [41]. Pork and chicken dishes were prepared by a commercial kitchen and delivered frozen to participants’ homes, and provided 28.1 ± 6.3 g and 25.2 ± 6.2 g of protein per meal, respectively. Muscle strength was assessed by GS, and lower extremity function by the sit-to-stand test, TUG, and 6-min walk test. Mean protein intake (g/kg BW) did not differ between the groups at baseline, 6 weeks, and 12 weeks post-intervention (1.11 ± 0.37 g, 0.97 ± 0.30 g, 1.06 ± 0.36 g, respectively). In multivariable adjusted linear mixed models, no differences in measures of muscle strength or function were found between the groups. For example, post-intervention GS in participants consuming pork was 25.7 ± 11.2 kg versus 26.2 ± 7.2 kg in those consuming chicken dishes. However, participants consuming chicken had improved verbal learning and memory test scores at six weeks (*p* < 0.001). The authors concluded that changing the type of dietary protein (pork versus chicken) had no impact on physical or mental function in older adults after a 12-week intervention [41].

In summary, only two independent studies examined the effect of protein-rich lean meat with [40] or without an exercise component [41] on muscle mass, strength, and function. Increased consumption of lean beef raising total protein intake to ~1.3 g/kg BW/day appeared to enhance the effect of resistance training on leg muscle strength and lean tissue mass in older women [40], the former substantially influencing their health-related quality of life [41].

##### Fruit and Vegetables (FV)

In the 16-week Ageing and Dietary Intervention Trial, 83 healthy community-dwelling older adults aged 65 to 85 years from Belfast, Northern Ireland who were habitually consuming ≤2 portions of FV a day were randomised to the intervention (≥5 portions of FV/day) and control group (≤2 portions of FV/day) to investigate the effect of an increased FV consumption on muscle strength (GS) and physical performance (SPPB) [43]. FV of participants’ choice were delivered to participants’ homes, and the compliance with FV intake monitored at baseline, 6, 12, and 16 weeks using diet histories interviews and laboratory assessment of biomarkers of micronutrient status. The study observed no significant differences in change in physical performance between the groups over 16 weeks, but a trend for a greater change in muscle strength (GS) in the ≥5 FV portions/day group compared with ≤2 FV portions/day group (mean change: 2.04 ± 5.16 kg versus 0.11 ± 3.26 kg, *p* = 0.06). Reanalysis using a general linear model and adjusted for change in physical activity confirmed the effect of FV on GS (*p* = 0.03). Increasing FV consumption from ≤2 portions/day to recommended levels was positively associated with the change in GS in between-groups analysis. Specifically, an extra daily portion of FV predicted a 0.5 kg (95% CI: 0.07–0.90) increase in GS, but no improvements were observed in SPPB. However, except for vitamin C the study showed a weak association between the change in micronutrient biomarkers status and GS. The study’s findings suggest that higher intake of FV (≥5 portion/day) may result in a modest increase in muscle strength in healthy older adults [43].

##### Multiple Whole Foods

In a 14-week RCT, 21 older men aged 65 ± 5 years were instructed to consume lacto-ovo vegetarian (LOV) diet for two weeks followed by a 12-week resistance training (RT) three times a week during which 10 participants were allocated to a beef diet and 11 participants to a LOV diet with texturized vegetable protein (soy products) [44]. Both diets providing 0.6 g of protein/kg BW a day from different protein sources (beef-containing diet: cube steak, ground beef, and beef tips; texturized soy products: vegetable sausage, vegetable hot dog, and vegetable chicken (Morningstar Farms, Worthington, OH, USA)). Beef diet had 57 ± 10% of total protein from supplemental beef, and LOV diet had 53 ± 7% of total protein from the supplemental soy products. The study examined whether the consumption of a meat-containing diet compared with a LOV diet influenced body composition and muscle strength in older men in response to a 12-week RT. Mean total protein intake over the 12-week RT was 1.03–1.17 g/kg BW/day. Men in both groups improved in maximum dynamic strength (14%–18%) in all muscle groups, but no differences between the groups were observed. Cross-sectional muscle area of vastus lateralis increased with both diets, but the differences between the diets were not significant (beef: 6.0 ± 2.6%; LOV: 4.2 ± 3.0%). Additionally, no differences were observed in body composition, and concentrations of muscle metabolites (creatine, phosphocreatine, and total creatine). The results indicated that the main source of dietary protein (beef or soy) when consumed in the amounts of ~1.1 g/kg BW/day did not preferentially influence the increase in muscle size and strength in older men during 12-week RT.

In a follow-up study, Haub et al. (2005) investigated the effect of beef versus texturized protein supplementation (soy) on lipoprotein-lipid concentrations, upper and lower muscle strength, and power after 12-week RT [45]. Post-intervention, men consuming the beef diet had an increased intake of saturated fat, cholesterol, zinc, and decreased intake of fibre resulting in higher levels of lipids/lipoproteins blood markers (HDL-cholesterol, LDL-cholesterol, and total cholesterol) compared with those in the LOV group. However, no change in cholesterol/HDL-cholesterol ratio was observed over the study period, which has been regarded as a more powerful predictor of cardiovascular health than individual lipids. Each group increased in upper and lower body strength, but there were no differences in strength gains between the groups, and no between-group difference in upper or lower body power output post-intervention. In conclusion, contrary to the hypothesis that soy protein may attenuate muscle protein metabolism in older men, the results demonstrated that the improvements in muscle strength and power were similar in the beef and LOV group during the 12 weeks of RT, suggesting that a LOV diet with resistance exercise may support gains in muscle performance in older adults equally well as diets with protein from animal sources [45].

In summary, we have identified only two studies that used either animal (lean beef) or plant-based protein source (soy products) added to a LOV diet showing no preferential effect of either whole food on muscle mass and function in older men.

##### Dairy (including Semi-Solids and Cheese)

Two RCTs used the same intervention protocol to investigate whether adding ricotta cheese (RCH) to the habitual diet (HD) may increase muscle mass, strength (GS), and physical performance in sarcopenic [46] and non-sarcopenic [47] Mexican older adults aged ≥ 60 years living in the community. In the first study, 40 sarcopenic older adults (23 women and 17 men; mean age 76 ± 5.4 years) were randomised into the intervention (HD + RCH) and control (HD) group. The intervention group consumed 210 g of ricotta cheese a day (15.7 g protein or 8.6 g/day EAA; 267 kcal/day extra energy) with the HD for 3 months. Both groups were instructed to resume their regular levels of physical activity. The primary aim of the study was to investigate the percent relative change in total appendicular skeletal muscle mass (TASM) and strength pre- and post-intervention across the groups. No significant difference in the percent relative change in TASM was observed between the groups after 3 months (2.2% in the intervention group versus 1.5% in controls), and GS improvements in the intervention group were not statistically significant (*p* = 0.06). However, in sex-specific analyses men in the HD + RCH group experienced significant gain in TASM compared to controls (490 g versus 220 g, respectively), and improved muscle strength (3.4% versus −0.3%), and lean body mass in arms (4.7% versus 1.7%). Thus, adding nutrient-rich dairy food (ricotta cheese) to a habitual diet may be beneficial in sarcopenic older men for gains in muscle mass and strength.

In the second study with the ricotta cheese intervention, 100 non-sarcopenic older adults (50 women and 50 men; aged 70.2 ± 7.0 years) were allocated to the intervention group (210 g ricotta cheese + HD) and control (HD) for 12 weeks to investigate the effect of dairy protein from cheese on TASM, GS, SPPB, and the stair-climb power test (SCPT) [47]. Participants who had TASM two standard deviations below the mean value of TASM for healthy Mexican population were excluded from the study. The relative change in TASM was positive in the intervention group (0.6 ± 3.5 kg) and negative in controls (−1.0 ± 2.6 kg) (*p* = 0.009). The percent relative change in GS in both groups were negative, but significantly more pronounced in the control group (−0.6 ± 10.8 versus −4.6 ± 10.8, *p* = 0.07). Although the relative change in balance test scores was positive in the RCH + HD group (3.7 ± 17.1 s) and negative in controls (−2.4 ± 12.7 s), the difference did not reach statistical significance (*p* = 0.05). No significant changes in SPPB and SCPT were observed. The authors concluded that the addition of ricotta cheese, a rich source of protein and other nutrients, to a habitual diet may improve appendicular lean muscle mass and balance, and may attenuate the loss of muscle strength in non-sarcopenic older adults [47].

In summary, only two studies used semi-solid/solid dairy (ricotta cheese) as a nutritional intervention to improve or attenuate the loss of muscle mass and strength in healthy [46] and sarcopenic older adults [47]. In both, adding one portion of ricotta cheese to a habitual diet was associated with gains in muscle mass and strength in sarcopenic and non-sarcopenic older adults.

##### Eggs

Wright et al. (2018) investigated the effect of a high protein diet (HP) on muscle composition, cardiometabolic health, and systemic inflammation in overweight and obese older adults [48]. Twenty-two participants (12 men and 10 women) aged 70 ± 5 years with BMI of 25–38 kg/m^2^ were allocated to the isocaloric HP with three whole eggs/day (1.4 g of protein/kg BW/day) or normal protein diet (0.8 g/kg BW/day) void of eggs (NP) for 12 weeks. Eggs/eggs products provided ~50 g/day (60%) of the additional dietary protein in the HP compared with the NP diet group distributed over breakfast, lunch, and mid-afternoon snack. No effects on muscle-specific composition were observed over time, independent of protein intake. Although total BW was reduced in both groups (−3.3 ± 1.2%) after 12 weeks, lean body mass was preserved in the HP group. Specifically, the HP diet prevented the decrease in trunk and total lean mass, which was significantly pronounced in the NP diet. Both diets decreased appendicular fat mass, but greater decreases were observed in in the NP diet (*p* = 0.03). Hip circumference and LDL decreased in the NP group over the study period, and no other changes in muscle composition (including intermuscular adipose tissue (IMAT), medial muscle cross-sectional area, muscle volume of thigh and calf muscle), cardiovascular health parameters or systemic inflammation (e.g., high sensitivity C-reactive protein, and insulin-like growth factor 1) were observed across the groups. The exception was the subcutaneous fat to muscle volume ratio at mid-calf, which significantly decreased in the HP group (*p* = 0.03). The results revealed no effect of a 12-week nutrition intervention with whole eggs on muscle composition, cardiovascular health or systemic inflammation in overweight and obese older adults [48].

### 3.4. Summary of Findings

#### 3.4.1. Whole Foods and Muscle Health in Later Life: Summary of Observational Studies

##### Myoprotective Whole Foods for Muscle Health

Based on evidence from 19 observational studies, the following whole foods appeared to be myoprotective in older adults (Table 3). Higher lean beef intake was associated with muscle mass [21,22,36] (e.g., each 100 g beef intake/week was associated with 0.1–0.13 increase in appendicular skeletal muscle index in non-obese, active older adults [22]; ≥2 servings/day of red meat (beef, lamb, pork; 1 serving = 28.35 g) was associated with 1.2% increase in skeletal muscle mass compared with <0.85 servings/day in women [36]).

A higher total intake of fruit and vegetables was associated with improved lean mass [24] in men, and physical performance in both men and women [25] (e.g., 2 to ≥5 portions of FV was associated with 60%–70% lower odds of slow walking speed compared with ≤1 portion [25]).

Consumption of 1 to ≥3 portion of fruit/day (1 portion = 120 g) was associated with 40%–50% reduced risk of slow walking speed compared to no intake [25] in older adults, whilst consumption of ≥2 servings/day of fruit (1 serving = 150 g) was associated with 30% decreased risk of low GS compared with <1 serving/day in older women [27]. Furthermore, high fruit intake was independently associated with 60% reduced odds of sarcopenia compared with low intake [24,28], and higher consumption of fruit and berries at age of 61 years was positively associated with Senior Fitness Test in women [35].

Consumption of 1 to ≥3 portions/day (1 portion = 150 g) of vegetables was associated with 30%–50% lower risk of slow walking speed compared with no intake in all participants [25]. In older women, ≥3 serve/day of vegetables (1 serving = 75 g) was associated with 31% reduced odds of low GS and TUG compared to <2 servings/day [27]. Furthermore, higher vegetable intake was associated with better chair rise and gait speed [30], and meeting the recommended ≥5 servings/day of vegetables versus not meeting the recommendations was associated with 50% lower risk of low muscle mass in women [31]. Furthermore, every day versus once every 2 days intake of green or yellow vegetables was associated with 60% lower decrease in knee extension strength in women [32].

Each extra portion of fatty fish was associated with ~0.5 kg of higher muscle strength (GS) in older adults [29], and higher intake of fish (white, shell, and oily) was associated with better gait speed in women [34]. Also, ≥3 servings/day of fish was associated with 1.2% extra mean skeletal muscle mass compared to older adults consuming <1 serving/day (1 serving = 28.35 g, cooked) [36]. Consuming soy products every day versus 1 per 2 days or less was associated with 70% lower decrease in knee extension strength in older women [32]. Higher Nordic cereal (rye, oats, and barley) intake at the age of 61 years was positively associated with Senior Fitness Test in both men and women 10 years later [35].

In women, dairy consumption of ≥2.2 servings a day (including yogurt and cheese) was associated with greater whole body lean mass, ASM, GS, and 30% lower odds of slow TUG compared with those consuming ≤1.5 serving/day [37,38]. Furthermore, older adults consuming ≥7 servings of dairy (including low-fat yogurt) had 40% lower risk of slow walking speed [39].

##### Detrimental Whole Foods for Muscle Health

The following whole foods were detrimental for muscle health in observational studies with older adults. A 100 g/day higher intake of processed meat was associated with poorer agility and 32% increased risk of lower extremity physical performance [23]. Japanese women consuming sea food every day compared to those consuming it once every 2 days or less had 1.5 times greater decline in knee extension strength over time [32].

#### 3.4.2. Whole Foods and Muscle Health in Later Life: Summary of Intervention Studies

##### Myoprotective Whole Foods for Muscle Health

Based on evidence from nine intervention studies [40,41,42,43,44,45,46,47,48] (six original RCTs, two follow-up studies, and one quasi-experimental pilot study), the following whole foods showed myoprotective properties (Table 3).

Lean red meat (beef, veal, and lamb) providing ~1.3 g of protein/kg BW/day (80 g servings of meat twice a day; ~45 g of total protein) for 4 months (6 days a week) combined with progressive resistant training performed twice a week may be beneficial for body composition, lower-body strength [40], and physical aspect of health-related quality of life [42] in older women. There was a 0.5 kg gain in lean tissue mass (0.3 kg in legs), and a 0.5 kg decrease in total fat mass, and a 18% increase in leg muscle strength [40], with latter being associated with overall health-related quality of life [42] in women consuming lean red meat compared to those in control group consuming ≥1 serving of pasta or rice (~75 g cooked; total protein: 1.1 g/kg BW/day).

Higher intake of fruit and vegetables (≥5 portions a day) over 16 weeks showed a trend in grater change in muscle strength (GS) compared with older adults consuming ≤2 portions of fruit and vegetables a day. An extra daily portion of fruit and vegetables predicted a 0.5 kg increase in grip strength [43].

A LOV diet supplemented with either beef dishes or texturized soy products providing mean protein intake of 1.03–1.2 g/kg BW/day or 53%–57% of total protein for 14 weeks in combination with a 12-week resistance training three times a week may equally support gains in muscle size, overall muscle strength [44], upper and lower body strength and power in older men [45].

Supplementing a habitual diet with 210 g of ricotta cheese a day for 3 months resulted in a significant gain in total appendicular skeletal muscle mass (460 g versus 220 g), grip strength (3.4% versus −0.3% relative change), and lean mass in arms (4.7% versus 1.7%) in sarcopenic older men compared with those without supplementation [46]. Daily supplementation of a habitual diet with 210 g of ricotta cheese for 12 weeks may improve appendicular muscle mass and balance, and attenuate muscle strength loss (grip strength) in non-sarcopenic older adults [47].

Enhancing a habitual diet with three whole eggs providing ~50 g of additional protein a day (total protein intake of 1.4 g/kg BW/day) compared with a normal protein diet (0.8 g/kg BW/day) for 12 weeks may preserve lean body mass (trunk and whole body), and decrease the subcutaneous fat to muscle volume ratio in overweigh/obese older adults [48].

### 3.5. Myoprotective Whole Foods in Later Life: Strongest Evidence

**Figure 2:**
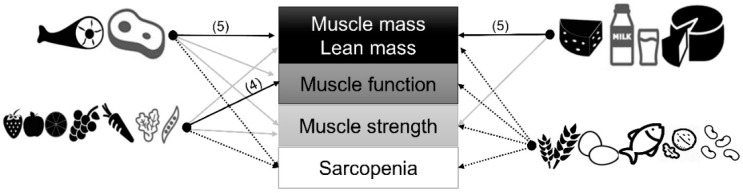
Myoprotective foods and muscle-related outcomes: summary of evidence. Summary of authors’ evaluation of the strength of evidence for the role of whole foods in muscle health in older adults in observational and intervention studies. The strength of evidence was determined based on the number of studies reporting statistically significant associations or effects, and the study quality (risk of bias assessments). The synthesis of findings showed strong and consisted evidence for the role of lean red meat and total dairy (including non-liquid dairy) in muscle mass or lean tissue mass in both observational and intervention studies (black bold line; red meat: 5 studies [21,22,36,40,45] (3 low, 1 medium, and 1 high risk of bias); dairy: 5 studies [37,38,39,46,47] (3 low, and 2 medium/somewhat risk of bias)). Higher intake of fruit and vegetables was associated with better muscle function in observational studies (black bold line; 4 studies [25,27,30,35] (2 low and 2 medium risk of bias)). There was moderate evidence for the role of these foods in muscle strength and sarcopenia (grey bold line; red meats: 3 studies [40,44,45] (1 low and 2 high risk of bias); fruit and vegetables: 4 studies [26,27,37,43] (2 low, 1 medium/somewhat, 1 high risk of bias), and dairy: 3 studies [37,46,47] (2 low and 1 medium/somewhat risk of bias)), and limited or inconclusive evidence for the benefits of other whole foods (e.g., cereal, fish, soy products) for muscle health in older adults (dashed black line; 9 studies [29,30,32,34,35,41,46,47,48] (3 low, 5 medium/somewhat, and 1 high risk of bias)). Several studies have used multiple whole foods and muscle-related outcomes.

Based on evidence synthesis (Table 1, Table 2 and Table 3) and risk of bias assessment (Table S3 and Figure S1) of 28 articles, the following whole foods were myoprotective (Figure 2). There was strong and consisted evidence for the role of lean red meat for skeletal muscle mass or lean tissue mass in both observational and intervention studies [21,22,36,40,45], and higher intake of fruit and vegetables for better muscle function in observational studies [25,27,30,35]. There was consistent evidence for the role of dairy (semi-solid and cheese) for appendicular muscle mass in observational and intervention studies [37,38,46,47]. However, there was only moderate evidence for the influence of these foods in muscle strength (red meats: [40,44,45], fruit and vegetables: [26,27,37,43] and mostly in women; dairy: [37,46,47]), and limited or inconclusive evidence for other whole foods (e.g., white meat, fish, eggs, soy products, cereal, and nuts) in relation to sarcopenia and other muscle-related outcomes [29,30,32,34,35,41,46,47,48].

## 4. Discussion

The aim of this systematic review was to evaluate which whole foods (meats, fish, eggs, fruit and vegetables, non-liquid dairy, and other foods (cereal, legumes, and nuts)) may be myoprotective in older adults aged ≥ 50 years. To our knowledge, this is the first evidence summary of observational and intervention studies that investigated these foods in relation to muscle health in older adults. We included 28 studies (19 observational and 9 intervention studies) that varied by the type of exposure/intervention, follow-up/duration, participants’ age, gender, sample size, and muscle-related outcomes. Thus, the findings were summarised descriptively, and their strength established based on the significant analytic results and the risk of bias assessment for each study. Complemented by other recent systematic reviews about the efficacy of liquid milk for muscle health [16], muscle anabolic response to protein-rich whole foods [52,53,54], total dairy protein [14,55], and protein supplementation [56] for muscle mass, strength and physical performance in older adults, this review focused on other nutrient-rich whole foods that may be beneficial for ageing muscle. Of 28 studied included in the review, 13 were judged to have low, 11 medium/some, and 4 high risk of bias. The synthesis of findings showed strong and consistent evidence for the benefits of lean red meat [21,22,36,40] and non-liquid dairy [37,38,39,46,47] in both observational and intervention studies on muscle mass or lean tissue mass. Higher intake of fruit and vegetables (separate and together) was associated with better muscle strength and function in observational studies [25,26,27,30,32,35], but the evidence from intervention studies was scarce [44]. Moderate evidence was observed for the role of meats, fruit and vegetables, and non-liquid dairy in muscle strength and sarcopenia [26,27,28,32,37,40,44,45,46,47]. There was either limited or inconclusive evidence for the benefits of other whole foods (e.g., cereal [35], fish [29,34,36], soy products [32,44,45], eggs [48]) for muscle health and function in older adults.

Recent increased scientific interest in a whole food rather than an isolated nutrient approach [52,53,54,55] to identify myoprotective foods is based on the premise that whole foods provide benefits that are greater than a sum of their constituents, acting synergistically and cumulatively upon ageing muscle. Whole foods contain nutrients and non-nutrients that may facilitate nutrient-nutrient interactions, nutrient behaviours, and act as signalling molecules for muscle anabolism and myoprotection [14,16,17,52]. Employing a food-first approach with older adults who understand foods better than isolated nutrients for healthy nutrition, and identifying those that are beneficial for muscle, will help in designing healthy eating patterns for overall and muscle health.

### 4.1. Protein-Rich Whole Foods and Ageing Muscle: Meats and Non-Liquid Dairy

A number of studies in this review have examined the role of protein-rich foods (meats, dairy, soy products, and eggs) for ageing muscle, emphasising the importance of higher protein intake to maintain and improve muscle mass and function in older adults either with or without exercise (e.g., [40,44,45,46,47,48]). In both observational and intervention studies, the authors hypothesised that increasing the protein intake above the recommended dietary allowance of 0.8 g/kg BW/day by consuming high-quality protein foods may result in increased muscle protein synthesis (MPS) and remodelling post-exercise. The higher protein needs of older compared with young adults have been extensively debated and researched, including in relation to the best protein source, quality and timing (e.g., [52,53,57,58,59,60]) to counteract a blunted anabolic response to protein feeding in ageing muscle. For example, Daly et al. (2014) used 160 g of lean red meat (beef, veal, and lamb) twice daily (~45 g of protein; ~15 g essential amino acids (EAA)) in combination with resistance exercise twice a week to achieve protein intake of ~1.3 g/kg BW on most days in older women. The study showed a 0.5 kg increase in lean tissue mass and 18% increase in muscle strength, contributed to higher protein intake [40]. Lean red meat has a balanced proportion of all eight amino acids required by older adults (including leucine for muscle protein synthesis), similar to concentrations observed in human muscle [61], and has been shown to stimulate MPS in middle-aged and older adults in dose-dependent manner [62]. A subject of much debate about health benefits and environmental impact, lean red meat contains other nutrients aside from protein, which may enhance muscle health, including unsaturated fatty acids, iron, vitamin D, magnesium, and zinc (reviewed in [63,64]). Equally, as a part of healthy, balanced diets, such as the Mediterranean diet, lean red meat contributes to nutrient adequacy of a range of nutrients for general heath in older adults [63], thus indirectly supporting healthy muscle ageing.

Of all protein sources and supplements for muscle health, dairy products, and isolated dairy proteins have gained the most attention [65]. In this review, non-liquid dairy (yogurt and cheese) as a source of high-quality protein, especially whey, have been used in relation to muscle mass, function, and sarcopenia in five studies with healthy and sarcopenic older adults [37,38,39,46,47]. All studies reported some benefits of higher dairy intake for lean tissue mass and muscle strength. Several protective biological mechanisms for muscle may be activated by dairy consumption. We have recently explored potential myoprotective properties of nutrients in dairy, including anabolic, anti-inflammatory, anti-oxidative, and immunomodulating potentials of proteins and derived bioactive peptides, fats and fatty acids, vitamins, and minerals in milk [16]. For example, milk products are rich in whey, which is abundant in branched-chain amino acid leucine, regarded to be superior to other amino acid for stimulating MPS through the rapamycin (mTOR) pathway [66]. In addition, a number of peptides (e.g., β-lactoglobulin, lactoferrin), lipids and fatty acids in milk fat globule (e.g., α-linoleic acid), and minerals (e.g., zinc) may help in neutralising reactive oxygen and nitrogen species (i.e., oxidative stress) implicated in pathophysiology of sarcopenia [67]. Furthermore, other nutrients in dairy such as casein-derived bioactive peptides and polyunsaturated fatty acids (e.g., n-3 PUFA) [12,13] may act against the chronic low-grade inflammation and cytokine load— another pathological mechanism of sarcopenia [68,69].

In summary, protein foods from animal sources (lean red meats, dairy products, and eggs) have been used in a number of studies with older adults to enhance daily dietary protein intake above the basic (0.8 g/kg BW/day) or sub-optimal levels (<0.8 g/kg BW/day) to preserve or increase lean muscle mass and function, alone or in combination with exercise. However, beyond anabolic stimulus, high-quality protein foods provide a range of other nutrients and non-nutrients with potential anabolic and other myoprotective properties, which are rarely considered, especially in interventions with older adults [52]. A recent review article by Burd et al. (2019) has emphasised “a need to define how dietary protein action on MPS rates can be modulated by other nutrients within a food matrix to achieve protein requirements for optimal muscle adaptations” (page S59) [52]. Specifically, our current understanding about the amount of dietary protein required for MPS post-exercise is based on the intake of isolated protein sources, and not on protein-rich whole foods. Because whole foods provide other nutrients and bioactive components, their potential anabolic and other biological effects on ageing muscle warrant further investigation.

When considering the best protein whole foods for lean muscle mass and function, aside from protein quantity, protein quality (e.g., digestibility/absorption rate to elicit MPS; protein quality scores) continues to be debated in light of muscle physiological outcomes, sustainability, and acceptability in older adults. Regarding physiological outcomes, a recent meta-analysis of studies including younger and older adults has shown no differences between animal protein (beef) versus dairy (whey) supplementation in increasing protein intake and lean tissue mass post-exercise [65]. Plant (e.g., pea and soy product) versus animal protein foods (e.g., dairy (whey)) have shown comparable effects on several muscle-related outcomes, including muscle thickness, force production [70], muscle size, strength [44], and power [45] in young and older adults. Several recent systematic reviews and opinion articles have provided a comprehensive overview about the importance of protein quantity and quality (animal versus plant) for healthy muscle in older adults and across the lifespan [15,53,54,57]. A healthy diet should include a variety of protein-rich whole foods to ensure nutrient density and diet quality in order to maximise health benefits of all nutrients and non-nutrients in the population within sustainable food systems (discussed in [53]). Regarding acceptability of protein-rich foods for muscle health, a feasibility study of two types of dietary advice aimed to increase dietary protein intake to 1.2 g/day per body weight adjusted to a healthy BMI has shown that both ‘even’ (a maximum of 20 g of protein per meal and snack occasion) and ‘peak’ (at least one daily meal of 35–45 g of protein) feeding strategies were acceptable to community-dwelling older adults aged ≥ 65 years [71].

### 4.2. Fruit and Vegetables and Ageing Muscle

The present evidence synthesis has shown a positive association between higher fruit and vegetables intake (separate and together) and several measures of muscle strength and function (e.g., walking speed, chair rise, TUG, Senior Fitness Test, grip strength, and leg extension strength) in eight observational studies [24,25,27,28,29,30,31,32]. Benefits of higher intake were consistent in older women (e.g., [27,30,32,35]). Although the exacts mechanism through which fruit and vegetables may provide myoprotection is not known, a number of constituents in fruits and vegetables have anti-oxidative and anti-inflammatory properties, potentially enhancing muscle function. Specifically, cumulative and synergistic effects of nutrients and non-nutrients in fruit and vegetables (e.g., fibre, vitamins, minerals, plant sterols, polyphenols, flavonoids, and alkaline salts) may increase the total exogenous anti-oxidative capacity in a healthy diet, and counteract pro-inflammatory response in ageing muscle. In addition, the phytochemical content of fruits and vegetable has been shown to improve cardiovascular and cardiometabolic health through several proposed mechanisms (e.g., antithrombotic, anti-atherosclerotic, lipid profile and blood pressure regulation, and glucose metabolism) [72], thus indirectly benefiting muscle health. However, because of well recognised limitations of observational studies (e.g., confounding and lack of causality), the findings presented here need to be interpreted with caution and corroborated in intervention studies with whole foods. To our knowledge, only one intervention study assessed the relationship of higher fruit and vegetables intake (≥5 portions a day) and muscle strength, showing a trend for positive association. Further well-designed intervention studies are needed to establish the influence of fruit and vegetables and other less-researched whole foods (e.g., fish, cereals, and nuts) on ageing muscle.

### 4.3. Strengths and Limitations

This systematic review has several limitations. Our inclusion criteria and the study selection process may have omitted relevant studies, resulting in reporting bias. Because of the heterogeneity and methodological differences across the studies, we opted for a descriptive summary of the findings. Although we used the established tools to assess the quality of the studies (the Newcastle–Ottawa Scale and the Cochrane Risk of Bias Tool), evaluation bias could not be excluded. Varying study protocols and measures of exposures made the endorsement of a definite dosage of a particular whole food for muscle strength and function difficult. Food frequency questionnaires were the most common tools to assess diet in observational studies, which may have misclassified the exposure. Estimated strength of evidence for muscle health was mainly based on observational studies (e.g., fruit and vegetables). A strength of our review is that it is the first systematic evaluation of non-liquid whole foods for muscle health in older adults and it was conducted according to the PRISMA statement. Most of the measures used to assess muscle-related outcomes were validated and reliable tools routinely used in research, and increasingly seen in clinical settings.

## 5. Conclusions

Utilising a whole food approach, we investigated which whole foods (meat, fish, eggs, fruit, vegetables, and non-liquid dairy) may be myoprotective in older adults aged ≥ 50 years. Descriptive evaluation of 28 studies showed strong and consisted evidence for myoprotection achieved by higher intake of lean red meat for muscle mass in both observational and intervention studies. Higher intake of fruit and vegetables was beneficial for muscle function in observational studies, but the evidence from intervention studies was limited. Non-liquid dairy foods were beneficial for muscle mass in both observational and intervention studies. Moderate evidence was observed for the role of these foods in muscle strength and sarcopenia. Furthermore, limited or inconclusive evidence were shown for the benefits of other whole foods (e.g., white meat, fish, cereal, and soy products) for muscle health in older adults. Although current nutritional recommendations are often based on a single nutrient (protein, EAA) approach, further research about the roles of protein-rich and other foods in muscle health and function will allow for the development of guidelines that are based on whole foods, also highlighting the potential importance of non-protein nutrients within these foods for myoprotection in older adults.

## Figures and Tables

**Table 1:** Observational studies involving whole foods and muscle-related outcomes in older adults aged ≥ 50 years.

Ref.	Study Participants & SETTING	Study Design	Exposure	Outcome Measures	Summary of Main Findings
Asp et al. (2012) [21]	142 adults aged 60–88; community-dwelling and ambulatory; OH, USA;	CS	Beef intake in the past 12 months assessed by the Diet History Questionnaire;	Muscle mass; GS;	Beef intake (g/day) was positively correlated to muscle mass measured by mid-arm muscle area (R = 0.128, *p* = 0.030). From multiple linear regression analysis, a 1 oz/day (~28 g/day) increase in beef consumption predicted a 2.3 cm^2^ increase in mid-arm muscle area. GS was not correlated with beef intake.
Morris & Jacques (2013) [22]	2425 participants in the NHANES (2003–2006) aged ≥ 50 years; Boston, MA, USA;	CS	Beef intake estimated from 2 × 24-h recall;	Appendicular skeletal muscle (ASM) index assessed by DXA;	Each 100 g/week increase in beef intake was associated with a 0.10 (*p* = 0.04) and 0.13 (*p* = 0.006) point increase in AMS index in non-obese participants who engaged in vigorous aerobic and muscle-strengthening exercises, respectively.
Struijk et al. (2018) [23]	2982 participants aged ≥ 60 years in the Seniors-ENRICA cohort; Spain;	PS	Meat intake assessed by a validated computer-assisted face-to-face diet history at baseline (2008–2010);	Functional tasks assessed by the Roscow–Breslau scale (agility and mobility); SPPB (a median follow-up 5.2 years);	Those in the highest tertile of processed meat had a higher risk of impaired agility (HR = 1.33; 95% CI 1.08–1.64, *p* = 0.01), and lower extremity function (SPPB) (HR = 1.32; 95% CI 1.02–1.68, *p* = 0.04) compared to those in the lowest tertile. A 100 g/day increase in processed meat consumption was associated with a 23% higher risk of poor agility. No associations were found with red meats (of any kind) and poultry.
Fruit and vegetables					
Kim et al. (2015) [24]	823 men and 1089 women aged ≥ 65 years; the Fourth KNHANES (2008–2009); Korea;	CS	Fruit and vegetables from FFQ;	Sarcopenia defined as relative lean mass (height and fat mass-adjusted lean mass) assessed by DXA;	Vegetables, fruit and combined FV was significantly associated with reduced risk of sarcopenia in older men (*p* = 0.03, *p* = 0.01, *p* = 0.003, respectively). Men in the highest quintile of vegetables (OR = 0.48, 95% CI: 0.24–0.95), fruit (OR = 0.30; 95% CI: 0.13–0.70) and FV consumption (OR = 0.32; 95% CI: 0.16–0.67) had lower risk of sarcopenia compared to those in the lowest quintile. Women in the highest quintile of fruit intake had a lower risk of sarcopenia (OR = 0.39; 95% CI: 0.18–0.83) compared to those in the lowest quintile.
Garcia-Esquinas et al. (2016) [25]	Three cohorts: 1872 men and women aged 68.7 ± 6.4 years in the Seniors-ENRICA cohort, Spain; 581 participants aged 81.8 ± 4.1 years in Three-City (3C) Bordeaux, France; 473 participants aged 74.5 ± 5.8 years in the Integrated Multidisciplinary Approach Cohort (AMI), rural France;	PS	Fruit and vegetables assessed by either a validated computerised diet history (the Seniors-ENRICA) or FFQ (3C Bordeaux, AMI);	Gait speed (3-m walking speed test);	Decreased risk of slow walking speed with increasing portions of fruit consumed per day. An inverse dose-response relation was found between the baseline consumption of fruit and risk of slow walking speed.
Ribeiro et al. (2016) [26]	432 African Americans aged 59.2 ± 4.4 years at in the African American Health Study. Study reports findings from multiple waves obtained (up to 10 year later); St. Louis, MO, USA;	PS	Fruit and vegetables assessed by the Behavioral Risk Factor Surveillance System at wave 8 (2008);	Gait speed, GS, SPPB, LBFL (wave 4 (2004) and wave 10 (2010));	Longitudinally, higher vegetables intake different from carrots, potatoes or salad was independently associated with better outcomes for GS, while fruit juice was associated with worse changes over time for GS.
Sim et al. (2018) [27]	1429 women aged ≥ 70 years in the Perth Longitudinal Study of Aging in Women; Perth, Australia;	PS	Fruit and vegetables from FFQ (validated by the Cancer Council of Victoria);	GS, TUG;	Vegetables consumption resulted in superior muscular strength (GS) and physical function (TUG). A 13% (*p* = 0.01) decreased risk of low GS (<22 kg) for every 75 g of vegetable serving. A 12% lower odds of slow TUG for every 75 g/day increase in vegetable intake. High vegetables intake (≥3 servings/day) was associated with 31% lower odds of low GS and 31% lower odds of slow TUG (both *p* = 0.02) compared with low vegetables intake (<2 servings/day).
Koyanagi et al. (2020) [28]	14,585 older adults aged ≥ 65 years from low- and middle-income countries in the Study on Global Ageing and Adults Health (China, Ghana, India, Mexico, Russia, South Africa);	CS	Fruit and vegetables assessed by a question ‘How many servings of fruit and vegetables do you eat on a typical day?’ and calculated in quintiles (Q1–Q5);	Sarcopenia;	In unadjusted analysis, increased fruit consumption was associated with lower prevalence of sarcopenia in women (21% in Q1 (0 servings) versus 7.9% in Q5 (≥4 servings)). In adjusted analysis, Q5 was associated with lower odds of sarcopenia (OR = 0.60, 95% CI 0.42–0.84, *p* < 0.01) compared to Q1 in all participants, and in women (OR = 0.42, 95% CI 0.24–0.73, *p* < 0.01) and not in men. No associations were found for vegetables intake.
Multiple whole foods					
Robinson et al. (2008) [29]	1569 men and 1414 women aged 59 to 73 from The Hertfordshire Cohort Study; Hertfordshire, UK;	CS; RS	White fish and shellfish, fatty fish, breakfast cereals, fruit and vegetables, nuts, eggs, offal, and other meats) assessed by FFQ based on the EPIC study questionnaire;	GS;	In multivariate analysis adjusted for height, age, and birth weight, each additional portion of fatty fish consumed per week was associated with a gain in GS of 0.43 kg (*p* = 0.005) in men and 0.48 kg (*p* < 0.001) in women.
Martin et al. (2011) [30]	628 participants aged 63–73; Hertfordshire, UK;	CS	Fruit and vegetables, nuts, meat and meat dishes, white and shellfish and oily fish assessed by FFQ;	SPPB (3-m walk time, chair-rise test, one-legged balance test);	An inverse association between vegetables (*p* = 0.02), white and shell fish (*p* = 0.04), and oily fish (*p* = 0.007) and 3-m gait speed in women, which was not robust to adjustments. Higher nuts (*p* = 0.01) and vegetables intake (*p* = 0.02) was associated with shorter char-rise time in women. However, after adjustments only the association with vegetables remained robust.
Kim et al. (2015) [31]	1486 men and 1799 women aged ≥ 65 years in the Fourth and Fifth KHANES (2008–2011); Korea;	CS	Meat, fish, eggs and legumes, fruit and vegetables assessed by FFQ;	ASM adjusted for weight and assessed by DXA;	In women, consuming recommended levels of vegetables (≥5/day from a list of 12 vegetables (Chinese cabbage, radish, dried radish leaves, bean sprouts, spinach, cucumber, hot peppers, carrots, pumpkin, cabbage, tomatoes, mushrooms) was associated with 48% lower odds of lower ASM (OR = 0.52, 95% CI 0.30–0.89). No associations with any of the food groups were observed in men.
Kojima et al. (2015) [32]	575 community-dwelling women aged between 75–85 years; Itabashi Ward of Tokyo, Japan;	CS (2008); PS (2012)	Green and yellow vegetables, potatoes, fruit, soy products, seaweeds, seafood, meat, egg, and milk intake frequencies assessed by close-ended lifestyle questionnaire;	Isometric KES;	Cross sectional: No significant relationship between KES and frequency of food intake of the studied food groups. Prospective: Daily intake of soy products and green or yellow vegetables was protective of KES decline over 4 years. The decrease of KES (17.87 N) in participants who ate soy products almost every day was approximately 69% of that in those who ate soy products once in 2 days or less (26.06 N, *p* = 0.03). The decrease of KES (18.82 N) in participants who ate green or yellow vegetables almost daily was approximately 60% of that in those who ate these vegetables once in 2 days or less (31.46 N; *p* = 0.02). Those who ate seafood almost daily had a 1.5 times greater decrease in KES (24.68 N) than those who ate seafood once in 2 days or less (16.88 N, *p* = 0.02).
Perala et al. (2016) [33]	1,072 men and women (mean age 71 years) in the Helsinki Birth Cohort Study (born 1934–1944); Helsinki, Finland;	PS	Elements of the Nordic Diet Score: Nordic fruit and berries (apples, pears and berries), Nordic vegetables (tomatoes, cucumber, leafy vegetables, roots, cabbages and peas), Nordic cereals (rye, oats and barley), Nordic fish (salmon and freshwater fishes), red and processed meat assessed with a validate FFQ at mean age of 61 (2001–2004);	SFT (assessed at mean age of 71 years (2011–2013));	In women, high consumption of Nordic fruit and berries (*p* = 0.01), and Nordic cereals (*p* = 0.03) were positively related to the overall SFT score, whilst higher consumption of red and processed meat were inversely associated with the SFT score (*p* = 0.001). In men, high consumption of Nordic cereals was associated with better overall SFT score (*p* = 0.04).
Hai et al. (2017) [34]	834 community-dwelling older adults aged between 60–92 years from Chengdu, Sichuan province, China	CS	Grains/cereals, fruit and vegetables, eggs, fish/shrimp, nuts, meat (pork, beef, mutton, poultry), milk/milk products, legumes assessed by a validated FFQ and based on the Chinese Food Guide Pagoda;	Sarcopenia defined according to the AWGS criteria;	Women with sarcopenia had lower frequency per week of nut consumption than those without sarcopenia (mean (SD): 0.05 (0.22) versus 0.81 (2.11), *p* = 0.02). Higher frequency per week of nut consumption was significantly associated with sarcopenia (adjusted OR 0.72, 95% CI 0.53–0.99, *p* < 0.05). No significant difference in regard to any of food groups was observed in men (with or without sarcopenia).
Perala et al. (2017) [35]	1072 men and women (mean age 71) in the Helsinki Birth Cohort Study (born 1934–1944); Helsinki, Finland;	PS	Elements of the Nordic Diet Score: Nordic fruit and berries (apples, pears and berries), Nordic vegetables (tomatoes, cucumber, leafy vegetables, roots, cabbages and peas), Nordic cereals (rye, oats and barley), Nordic fish (salmon and freshwater fishes), red and processed meat assessed with a validate FFQ at mean age of 61 (2001–2004);	GS, isometric leg strength (knee extension), body composition (BIA) assessed at mean age of 71 years (2011–2013)	In women, Nordic cereals intake were positively related to leg strength change (*p* = 0.05), whereas red and processed meats were inversely related to GS change (*p* = 0.001).
Bradlee et al. (2018) [36]	1016 men and 1333 women median age 52 years in the Framingham Offspring Study; Boston starting in 1972, USA;	PS	Red meats, poultry and fish, dairy, legumes, soy, nuts and seeds assessed at exam 3 and 5 from 3-day dietary records; servings/day derived using the standard United States Department of Agriculture serving size;	Skeletal muscle mass (SMM) assessed by BIA at exam 6 and 7 (approximately 9-year follow-up); selected functional tasks reflecting impaired muscle function from the Roscow–Breslau scale and the Nagi scale assessed at exam 5 through 8 (approximate 13-year follow-up);	Women who consumed ≥2 servings of red meats (beef, lamb, and pork)/day had an extra mean 1.2% SMM (p<0.001) compared with those consuming <0.85 servings/day (1 serving = 1 ounce, cooked). Men and women consuming ≥3 servings/day of poultry and fish compared to those consuming <1 had an extra mean % SMM of 0.8 (*p* = 0.02) and 1.2 (*p* = 0.001), respectively (1 serving = 1 ounce, cooked). Men and women consuming ≥1.25 servings/day of legumes, soy, nuts and seeds compared with those consuming < 0.25 had an extra mean % SMM of 0.7 (*p* = 0.02) and 0.8 (*p* = 0.02), respectively over 9 years (1 serving = 1 cup, cooked). A non-significant 20% reduction in developing functional decline in ≥2 tasks in those aged ≥ 50 years who consumed ≥1 serving/day of dairy (1 serving = 1 cup of milk or yoghurt, 1–1.5–ounce cheese) or ≥1 (women) or ≥2 (men) servings of poultry and fish versus less.
Dairy (including semi-solids and cheese)					
Radavelli-Bagatini et al. (2013) [37]	1456 women aged 70 to 85 years in the Calcium Intake Fracture Outcome Study (CAIFOS); Western Australia;	CS	Dairy intake (milk, yogurt and cheese products) assessed by a validated FFQ at baseline in 1998;	Skeletal muscle mass assessed by DXA, GS, and TUG	Compared to those in the lowest tertile of dairy intake (≤1.5 serving/day), women in the highest tertile (≥2.2 servings/day) had significantly greater whole body lean mass (34.4 ± 03 vs. 32.9 ± 0.03 kg, *p* = 0.001), ASM (15.3 ± 0.2 vs. 14.5 ± 0.2 kg, *p* = 0.002), greater GS (20.9 ± 0.2 vs. 20.0 ± 0.2 kg, *p* = 0.02), and 26% lower odds of poor TUG (>10.2 s) (*p* = 0.04).
Radavelli-Bagatini et al. (2014) [38]	564 women aged 80 to 92 years in the Calcium Intake Fracture Outcome Study (CAIFOS) Aged Extension Study (CAIFOS/CARES); Western Australia;	CS	Dairy intake (milk, yogurt and cheese products) assessed by a validated FFQ from the Cancer Council Victoria at 10-year follow-up in 2008;	Skeletal muscle mass assessed by DXA	Women in the highest tertile of dairy intake (≥2.2 servings/day) had 4.0% higher ASM (*p* = 0.04) compared with the lowest tertile (≤1.5 serving/day), which remained significant after multivariate adjustments (3.3%, *p* = 0.01) (1 serving = 250 g milk, or 200 g yogurt, or 40 g of hard, firm, soft, and low-fat cheese, or 120 g cottage/ricotta cheese).
Lana et al. (2015) [39]	1871 adults aged ≥ 60 in the Study of Nutrition and Cardiovascular Risk (Seniors-ENRICA); Spain	PS	Dairy (milk, yogurt, cheese) assessed at baseline (2008–2010) using a validated diet history (developed form the European Prospective Investigation into Cancer and Nutrition);	Gait speed (defined as height and sex-adjusted lowest quintile in the sample), GS.	Those who consumed ≥7 servings/week of dairy (low-fat milk or yogurt) had lower risk of slow gait speed (OR = 0.64, 95% CI = 0.44–0.92, *p* = 0.01). No associations were found for GS. One standard serving was defined as 250 mL for milk, 150 mL for yogurt, and 40 g for cheese.

Abbreviations: ASM, appendicular skeletal muscle mass; AWGS, Asian Working Group for Sarcopenia; CI, confidence interval; CS, cross-sectional study; BIA, bioelectrical impedance; DXA, dual-energy X-ray absorptiometry; EPIC, European Prospective Investigation into Cancer and Nutrition study; FFQ, food frequency questionnaire; LBLF, lower body functional limitations; FV, fruit and vegetables; GS, grip strength; HR, hazard ratio; KES, knee extension strength; KHANES, Korea National Health and Nutrition Examination Survey; NHANES, National Health and Nutrition Examination Survey; PS, prospective study; Seniors-ENRICA, Study of Nutrition and Cardiovascular Risk Factors in Spain; SFT, Senior fitness test; SPPB, Short Physical Performance Battery; TUG, Timed Up-and-Go Test.

**Table 2:** Intervention studies involving whole foods and muscle-related outcomes in older adults aged ≥ 50 years.

Ref.	Study Participants & SETTING	Study Design	Intervention/Exposure	Outcome Measures	Summary of Main Findings
Meats					
Daly et al. (2014) [40]	91 women aged 60–90 years; Melbourne, Australia;	Cluster RCT	PRT twice a week and allocated to either 160 g/day (cooked) lean red meat consumed across 2 meals/day for 6 days/week or ≥1 serving/day (25–30 g) carbohydrates (pasta or rice) for 4 months;	LTM, FSST, TUG, STS;	The intervention group experienced great gains in total body LTM and muscle strength compared with control group. Increases were demonstrated in a 10% greater increase in serum insulin-like growth factor I and a 16% greater reduction in the proinflammatory marker.
Charlton et al. (2016) [41]	Healthy older people aged ≥ 60 years (n = 48); mean age 78.2 ± 6.2 years; New South Wales, Australia;	Quasi-experimental study	Participants were instructed to continue with their habitual diet but to substitute 4 meals/week with either (1) pork (intervention) or (2) chicken (control)-containing meals for 12 weeks;	GS, sit-to-stand test, get-up-and-go test and 6MWT;	Provision of 4 pork meals/week did not result in improvements in cognitive function, nor measures of muscle strength or physical performance, compared to those consuming chicken meals (control) in healthy older adults.
Torres et al. (2017) [42]	91 women aged 60–90 years; Melbourne, Australia;	Cluster RCT	PRT twice a week and allocated to either 160 g/day (cooked) lean red meat consumed across 2 meals/day for 6 days/week or ≥1 serving/day (25–30 g) carbohydrates (pasta or rice) over 4 months;	LTM, muscle strength (1-RM);	PRT combined with diet enriched with lean red meat induced changes in lower limb muscle strength not but LTM.
Fruit and vegetables					
Neville et al. (2013) [43]	80 healthy, community-dwelling older adults aged 65–85 years; Belfast, Ireland;	RCT	Participants randomised to continue their normal diet (≥2 portions FV/day), or to consume ≥5 portions of FV/day for 16 weeks.	GS, SPPB;	Increased FV to 5 portions/day resulted in a modest increase in GS, but no effect on physical performance (SPPB) in healthy older adults.
Multiple whole foods					
Haub et al. (2002) [44]	21 men (mean age 65 ± 5 years); AR, USA;	RCT	Men consumed habitual diets during the first week. During the next two weeks, all participants consumed a self-select LOV diet (textured vegetable protein (soy) products)). For the remaining 12 weeks, men were randomly assigned to either continue the LOV diet or begin a beef-containing diet (a self-selected LOV diet supplemented with beef);	Body density (plethysmography), biopsy, cross-sectional muscle area (computed tomography scans performed on a General Electric scanner);	There were no differences between the two groups in terms of muscle strength and size.
Haub et al. (2005) [45]	21 men (mean age 65 ± 5 years); AR, USA	RCT	Men consumed habitual diets during the first week. During the next two weeks, all participants consumed a self-select LOV diet, including textured vegetable protein (soy) products. For the remaining 12 weeks, men were randomly assigned to either continue the LOV diet or begin a beef-containing diet (a self-selected LOV diet supplemented with beef). RT 3 day/week during the 12-week period;	Muscle strength and muscle power (3 maximal repetitions at 4 intensities relative to their 1-RM at the time of resting);	There were no differences between groups for upper body or lower body output at baseline or 12 weeks post RT and no differences in strength gains.
Dairy (including semi- solids and cheese)					
Aleman-Mateo et al. (2012) [46]	Older women (n = 23) and men (n = 17) with sarcopenia, mean age 76 ± 5.4 years; Hermosillo, Sonora, Mexico;	RCT	Participants in the intervention group were asked to consume their HD but add 210 g of ricotta cheese a day over 3 months (HD + RCH; divided into three equal portions of 70 g, ingested at breakfast, lunch and dinner). Subjects in the control group were instructed to consume only their habitual diet;	TASM, GS;	No differences between groups for changes in TASM and GS. Sarcopenic men in HD+RCD gained 260 g of TASM compared with 220 g of TASM in control group (2.7 versus 1.2% relative change), and lean body mass in arms (4.7 versus 1.3% relative change).
Aleman-Mateo et al. (2014) [47]	100 healthy older men (n = 50) and women (n = 50) age 70.2 ± 7.0 years; Hermosillo, Sonora, Mexico;	RCT	Participants in the intervention group were asked to consume their HD but add 210 g of ricotta cheese a day over 12 weeks (HD+RCH; divided into three equal portions of 70 g, ingested at breakfast, lunch and dinner). Subjects in the control group were instructed to consume only their HD;	GS, SPPB, ASM;	Difference between groups for ASM (*p* = 0.009), intervention group (0.6 ± 3.5 kg) vs. control (−1.0 ± 2.6). No significant difference for GS, SPPB score, gait speed, and 5-chair rises, whilst balance scores were higher in intervention group.
Eggs					
Wright et al. (2018) [48]	22 adults aged 50–80 years; West Lafayette, Indiana, USA;	Parallel-design RCT	A 12-week diet with three whole eggs per day versus a HD void of eggs;	muscle composition (lean mass, IMAT, MSCA)	A 12-week high-protein diet with whole eggs did not improve muscle composition in older adults with overweight or obesity.

Abbreviations: ASM, appendicular skeletal muscle mass; FSST, 4-square step test; FV, fruits and vegetable; GS, grip strength; HD, habitual diet; IMAT, inter-muscular adipose tissue; LOV, lacto-ovo vegetarian; LTM, lean tissue mass; MSCA, muscle cross-sectional area; PRT, progressive resistance training; RCT, randomised control trial; RM, repetition maximum; RT, resistance training; SPPB, Short Physical Performance Battery; STS, 30-s sit-to-stand test; TUG, Timed Up-and-Go Test; TASM, total appendicular skeletal muscle; 6MWT, six-minute walk test.

**Table 3:** Myoprotective whole foods: summary of evidence from observational and intervention studies.

Whole Foods	Muscle Health Outcome	Who?	Study Design and Quality of Evidence	Ref.
Meats	Observational studies			
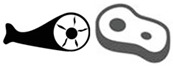	mid-arm muscle area	older adults	cross-sectional; low RoB	[21]
	ASM index	older adults	cross-sectional; low RoB	[22]
	SMM	women	prospective; medium RoB	[36]
Fruit and vegetables				
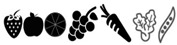	lean muscle mass	men	cross-sectional; medium RoB	[24]
	walking speed	older adults	prospective; medium RoB	[25]
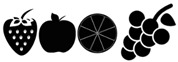	lean muscle mass	women	cross-sectional; medium RoB	[24]
	walking speed	older adults	perspective; medium RoB	[25]
	grip strength	women	prospective; low RoB	[27]
	sarcopenia	older adults, women	cross-sectional; low RoB	[28]
	physical performance (SFT)	women	prospective; low RoB	[35]
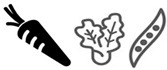	walking speed	older adults, women	prospective, cross-sectional; medium RoB	[25,30]
	grip strength	older adults, women	prospective; high & low RoB, respectively	[26,27]
	knee extension strength	women	prospective; low RoB	[32]
	physical performance (TUG)	women	prospective; low RoB	[27]
	chair rise	women	cross-sectional; medium RoB	[30]
	ASM	women	cross-sectional; low RoB	[31]
Dairy products				
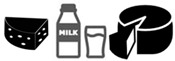	ASM	women	cross-sectional; low & medium RoB	[37,38]
	grip strength	women	cross-sectional; low RoB	[37]
	physical performance (TUG)	women	cross-sectional; low RoB	[37]
	walking speed	older adults	prospective; low RoB	[39]
Meats	Intervention studies			
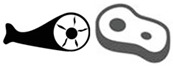	lean tissue mass	older women	RCT; low RoB	[40]
	muscle strength (leg extension)	older women	RCT; low RoB	[40]
	Physical Component Score of SF-36	older women	RCT; low RoB	[42]
Fruit and vegetables				
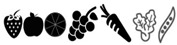	grip strength	older adults	RCT; high RoB	[44]
Supplemented LOV ^1^				
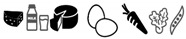	muscle strength (upper & lower body)	older men	RCT; high RoB	[44,45]
	muscle power	older men	RCT; high RoB	[45]
	muscle size	older men	RCT; high RoB	[45]
Dairy products				
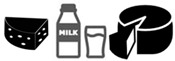	total ASM	sarcopenic men	RCT; some concerns for RoB	[46]
	lean body mass in arms	sarcopenic men	RCT; some concerns for RoB	[46]
	grip strength	sarcopenic men & healthy older adults	RCT; some concerns; low RoB	[46,47]
	lean tissue in legs	healthy older adults	RCT; low RoB	[47]
	total muscle mass	healthy older adults	RCT; Low RoB	[47]
Eggs				
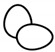	lean body mass (trunk and total)	overweight/obese older adults	RCT; some concerns for RoB	[48]

^1^ LOV supplemented with eithr beef or soy products. Abbreviations: ASM, appendicular skeletal muscle mass; LOV, lacto-ovo vegetarian diet; RCT, randomised control trials; RoB, risk of bias; SFT, Senior Fitness Test; SMM, skeletal muscle mass; TUG, Timed Up-and-Go Test.

## Supplementary Materials

The following are available online at https://www.mdpi.com/2072-6643/12/8/2257/s1, Table S1: Exclusion criteria for study selection, Table S2: Example of initial search terms and strategy for two databases, Table S3: Risk of bias assessment scores for observational studies, Figure S1: Risk of bias assessment for intervention studies.
