# Supplementary material for: Myoprotective Whole Foods, Muscle Health and Sarcopenia: A Systematic Review of Observational and Intervention Studies in Older Adults
Source: Nutrients. 2020 Jul 28;12(8):2257. doi: 10.3390/nu12082257 (PMC7469021; doi:10.3390/nu12082257)
Supplement: Supplementary file 1 [file nutrients-12-02257-s001.pdf]

Supplementary Materials

1. Supplementary Materials Content

Table S1. Exclusion criteria for study selection

Table S2. Example of initial search terms and strategy for two databases

Table S3. Risk of bias assessment for observational studies

Figure S1. Risk of bias summary for intervention studies

2. Supplementary Tables

Table S1. Inclusion and exclusion criteria for study selection

Parameter	Inclusion criteria	Exclusion criteria
Article type/study design	full text; observational studies (cross-sectional and longitudinal); RCT	reviews; trial protocols; conference abstracts; book chapters; studies without control/placebo group; case reports for RCTs; opinions; commentaries; editorials; mixed methods research articles for observational studies
Language	English	
Publication year/time frame	2000 until March 5, 2020	
Population	older adults aged 50 and over;<br>sample size: ≥50 participants for observational and ≥10 participants per group for RCT	
Setting	community; care homes; inpatient and outpatient facilities; hospitals	
Exposure	whole foods available to older adults (solid/semi-solid milk (yoghurt and cheese), meat, eggs, fish, fruits and vegetables) clearly described;<br>amount/dosage, and frequency/duration of consumption clearly defined;<br>intervention duration (a whole food with or without exercise component) at least 6 weeks	biomarkers of macro and micronutrient status (e.g., plasma carotenoids for fruits and vegetables consumption); liquid foods only (e.g., juices, liquid milk[1], liquid protein-based supplements); reconstituted liquid or semi-solid foods; fortified/enriched whole foods; whole diet/dietary pattern with no clear associations between individual foods and muscle-related outcomes
Primary outcomes	measures of skeletal muscle mass; muscle strength; muscle power; muscle function; physical performance (e.g., gait speed, Timed Up-and-Go test, Senior Fitness Test, chair stand test)	postprandial muscle protein synthesis; muscle fibers; muscle biopsy

[1]Studies with liquid milk only without other dairy products. Studies including several dairy products (solid/semi-solid dairy) along with liquid milk were included.

Table S2. Example of initial search terms and strategy for two databases

	Searches	Results	
		Medline	EMBASE
1	exp Muscle Weakness/ or sarcopenia.mp. or exp Muscle, Skeletal/ or exp SARCOPENIA/ or exp Muscular Atrophy/	180252	536562
2	("muscle mass" or " muscle strength" or " grip strength" or " walking speed" or " gait speed" or " appendicular lean mass" or " skeletal muscle index" or " physical performance" or " Timed Up-and-Go test" or " muscle wasting" or "age-related muscle loss" or " myopenia" or " dynapenia" or " sarcopenia" or "sarcopenic").tw.	38382	75778
3	1 or 2	203611	579533
4	exp Aged/ or exp "Aged, 80 and over"/ or exp Middle Aged/	2914357	3034826
5	("older adult" or elderly).tw.	139295	282881
6	4 or 5	2935247	3095887
7	food/ or exp cheese/ or exp yogurt/ or exp eggs/ or exp fruit/ or exp meat/ or exp vegetables/ or exp FISHES/	243273	494310
8	(food or cheese or yogurt or eggs or fruit or meat or vegetables or FISHES).tw.	319425	547861
9	7 or 8	482010	860522
10	3 and 6 and 9	610	1415
11	limit 10 to (english language and humans and yr="2000 -Current")*	532	1114
12	limit 11 to full text	139	244
13	limit 12 to "middle aged (45 plus years)" [Limit not valid in Embase; records were retained]	136	244

14	limit 13 to (observational study or randomized controlled trial) [Limit not valid in Embase; records were retained]	31	224
15	limit 12 to (adult <18 to 64 years> or aged <65+ years>) [Limit not valid in Ovid MEDLINE(R); records were retained]	31	224
16	limit 15 to article [Limit not valid in Ovid MEDLINE(R); records were retained]	**31**	**170**

[1]current: December 2018

Table S3. Risk-of-bias assessment scores for observational studies[1]

**Ref.**	**1. Study design**	**2. Study participants**	**3. Measurements of diet quality**	**4. Measurements of outcomes**	**5. Confounding factors**	**6. Blinding**	**7. Follow up**	**8. Info on non-participants**	**9. Analysis**	**10. Sample size**	**Overall quality rating: Risk of bias**
Asp et al., (2012) [22]	+1	0	+1	0	+1	0	+1	-1	+1	0	+4 = low
Morris & Jacques, (2013) [23]	+1	+1	0	0	+1	0	+1	-1	+1	+1	+5 = low
Struijk et al., (2018) [24]	+1	+1	+1	0	+1	+1	0	+1	+1	+1	+8 = low
Kim et al., (2015) [25]	+1	0	0	-1	+1	0	+1	-1	+1	+1	+3 = medium
Garcia-Esquinas et al., (2016) [26]	+1	+1	+1	-1	0	0	-1	-1	+1	+1	+2 = medium
Ribeiro et al., (2016) [27]	0	-1	-1	+1	-1	0	-1	-1	0	0	-4 = high
Sim et al., (2018) [28]	0	+1	+1	-1	0	0	+1	0	+1	+1	+4 = low
Koyanagi et al., (2020) [29]	+1	+1	-1	0	+1	0	+1	0	+1	+1	+5 = low
Robinson et al., (2008) [30]	+1	+1	0	-1	-1	0	+1	0	0	+1	+2 = medium
Martin et al., (2011) [31]	+1	+1	0	-1	0	0	0	-1	0	0	0 = medium
Kim et al., (2015) [32]	+1	0	0	+1	+1	0	+1	-1	+1	+1	+5 = low
Kojima et al., (2015) [33]	+1	+1	0	0	-1	0	0	0	+1	0	+1 = medium
Perala et al., (2016) [34]	+1	+1	0	0	-1	0	0	0	0	+1	+1 = medium
Hai et al., (2017) [35]	+1	+1	0	+1	0	0	+1	-1	+1	0	+4 = low
Perala et al., (2017) [36]	+1	+1	+1	+1	0	0	-1	0	+1	+1	+5 = low
Bradlee et al., (2017) [37]	+1	+1	-1	0	+1	0	-1	-1	+1	+1	+2 = medium

Radavelli-Bagatini et al., (2013) [38]	+1	+1	+1	+1	+1	0	+1	-1	+1	+1	+7 = **low**
Radavelli-Bagatini et al., (2014) [39]	+1	+1	+1	+1	+1	0	-1	-1	0	0	+3 = **medium**
Lana et al., (2015) [40]	+1	+1	0	0	0	0	0	0	+1	+1	+4 = **low**

[1]For each question a suitable quality score/risk of bias score was assigned: -1 (high), 0 (medium), and +1 (low). Total score range: -9 to +10; score of -9 to -3 = **high risk of bias**; -2 to +3 = **medium risk of bias**; +4 to +10 = **low risk of bias** based on the Newcastle-Ottawa screening tool for observational studies (http://www.ohri.ca/programs/clinical_epidemiology/oxford.asp).

3. Supplementary Figures

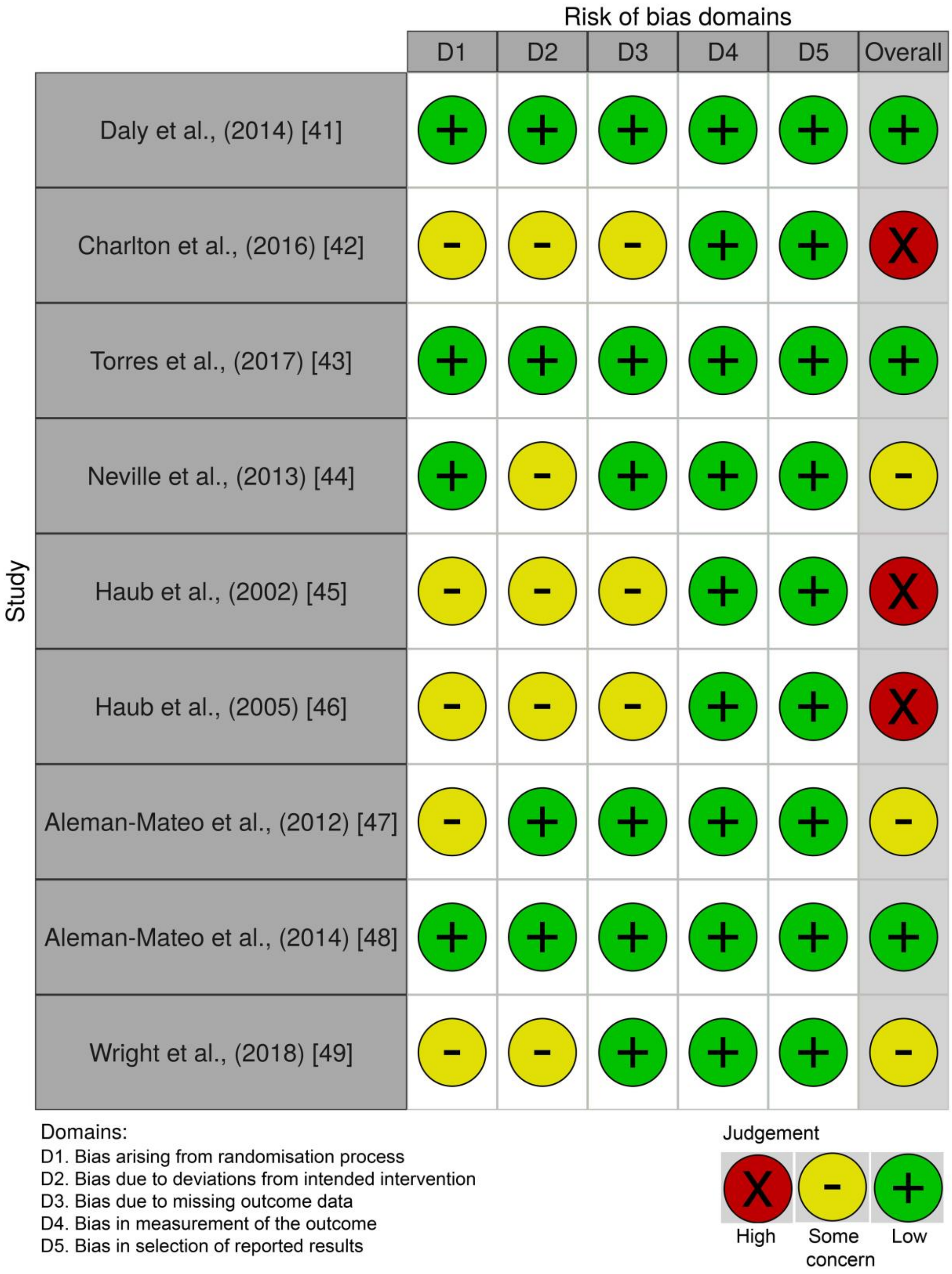

Figure S1. Risk of bias summary for intervention studies.

Review of authors' judgments for each risk of bias domain for nine included studies using the Cochrane risk of bias tool [21] comprising of five domains. Each domain contains 3–7 signaling questions, which are evaluated as 'low risk', 'high risk', and 'some concerns' for risk of bias. Overall risk of bias judgment was determined from the individual domains' risk of bias responses. Robvis[1] tool was used to create the graph.

[1]McGuinness, L.A.; Higgins, J.P.T. Risk-of-bias VISualization (robvis): An R package and Shiny web app for visualizing risk-of-bias assessments. *Res. Syn.Meth*. **2020**, 1- 7. https://doi.org/10.1002/jrsm.1411).

© 2020 by the authors. Submitted for possible open access publication under the terms and conditions of the Creative Commons Attribution (CC BY) license (http://creativecommons.org/licenses/by/4.0/).
